# Sequence differences in the seed dormancy gene *Qsd1* among various wheat genomes

**DOI:** 10.1186/s12864-017-3880-6

**Published:** 2017-06-29

**Authors:** Kazumitsu Onishi, Miki Yamane, Nami Yamaji, Mayumi Tokui, Hiroyuki Kanamori, Jianzhong Wu, Takao Komatsuda, Kazuhiro Sato

**Affiliations:** 1 Obihiro University of Agriculture and Veterinary Medicine, Obihiro, 080-8555 Japan; 2 Institute of Plant Science and Resources, Okayama University, Kurashiki, 710-0046 Japan; 3 Institute of Crop Science, National Agriculture and Food Research Organization, Tsukuba, 305-8634 Japan

**Keywords:** *Aegilops*, Alanine aminotransferase, Orthologs, Pre-harvest sprouting, Quantitative trait locus, *Triticum*

## Abstract

**Background:**

Pre-harvest sprouting frequently occurs in *Triticum aestivum* (wheat) and *Hordeum vulgare* (barley) at the end of the maturity period due to high rainfall, particularly in Asian monsoon areas. Seed dormancy is a major mechanism preventing pre-harvest sprouting in these crops.

**Results:**

We identified orthologous sequences of the major *Hordeum vulgare* (barley) seed dormancy gene *Qsd1* in hexaploid wheat cv. Chinese Spring by performing genomic clone sequencing, followed by transcript sequencing. We detected 13 non-synonymous amino acid substitutions among the three sub-genomes of wheat and found that the *Qsd1* sequence in the B sub-genome is most similar to that in barley. The *Qsd1* sequence in A genome diploid wheat is highly similar to that in the hexaploid A sub-genome. Wheat orthologs of *Qsd1* showed closer similarities to barley *Qsd1* than did those of other accessions in the DNA database. Like barley *Qsd1*, all three wheat *Qsd1s* showed embryo-specific gene expression patterns, indicating that barley and wheat *Qsd1* share an orthologous origin. The alignment of four hexaploid wheat cultivars indicated that the amino acid sequences of three spring cultivars, Chinese Spring, Haruyo Koi, and Fielder, are exactly the same in each sub-genome. Only Kitahonami has three amino acid substitutions at the B sub-genome.

**Conclusions:**

Kitahonami has a longer seed dormancy period than does Chinese Spring. Sequence polymorphisms between Chiniese Spring and Kitahonami in the B sub-genome may underlie the phenotypic differences in seed dormancy between these hexaploid wheat cultivars.

**Electronic supplementary material:**

The online version of this article (doi:10.1186/s12864-017-3880-6) contains supplementary material, which is available to authorized users.

## Background

Seed dormancy is one of the most important agronomic traits of small grain cereals growing in temperate climates. Pre-harvest sprouting frequently occurs in *Triticum aestivum* (wheat) and *Hordeum vulgare* (barley) at the end of the maturity period due to high rainfall, particularly in Asian monsoon areas. Seed dormancy is a major mechanism preventing pre-harvest sprouting in these crops.

Phytohormones, particularly abscisic acid (ABA), have been shown to regulate seed dormancy in several plant species [1–5]. In Arabidopsis, MOTHER OF FT AND TFL1 (MFT1) is directly regulated by ABA-INSENSITIVE3 (ABI3) and ABI5 and is upregulated by DELLA proteins in the GA signaling pathway [6]. Nakamura et al. [7] found that a homolog of MFT1, MFT, positively regulates seed dormancy in hexaploid wheat. Recent gene isolation studies have shown that other factors also regulate seed dormancy. Nakamura et al. [8] reported that *Qsd2*, encoding MAP3K (mitogen-activated protein kinase cascade), regulates seed dormancy in barley, with longer periods of dormancy found among East Asian landraces. Sato et al. [9] identified a major seed dormancy QTL, *Qsd1*, by analyzing the progeny of a cross between a wild ancestral form of barley and a cultivated barley. *Qsd1* encodes an alanine aminotransferase (AlaAT) family member, which has not hitherto been reported to function in dormancy in any plant species. Of the five *AlaAT* genes present in barley [9], only *Qsd1* is specifically expressed in embryos at maturation. A homolog of *Qsd1* in rice also shows embryo-specific gene expression, but has not been shown to be responsible for seed dormancy. Thus, the main function of AlaAT appears to be other than the control of seed dormancy [10, 11].

Several studies have focused on seed dormancy in wheat. The major QTLs controlling seed dormancy were found on chromosome 3AS (*QPhs.ocs-3A.1*) [12] and chromosome 4AL (*Phs-1*) [13], respectively. *Phs-1* was identified as an orthologue of barley *Qsd2* [14]. Nakamura et al. [7] mapped MFT on chromosome 3A, which co-localizes with the seed dormancy QTL *QPhs.ocs-3A.1* [12]. Hori et al. [15] identified a major QTL on chromosome 5 using a mapping population of einkorn wheat (*T. boeoticum* x *T. monococcum*) that might be an ortholog of barley *Qsd1*. However, to date, barley *Qsd1* orthologs have not been identified or characterized in hexaploid wheat.

Seed dormancy in barley and wheat is often controlled by recessive genes [7, 8, 15–17]. These genetic components might have developed via human selection for dominant, non-dormancy mutations during the pre-domestication period and subsequent cultivation/utilization processes after domestication. Since non-dormancy mutations might occur frequently during cultivation, it is difficult to identify an entire seed dormancy QTL in a species.

Since hexaploid wheat (*T. aestivum*) has three sub-genomes, A, B, and D, the contributions of these sub-genomes to a trait are difficult to estimate in most cases, since non-functional alleles in a sub-genome cannot be observed as a phenotype in a plant. On the other hand, it is also possible to collect non-functional (recessive) alleles from every sub-genome in order to establish a novel phenotype, as demonstrated by developing a hexaploid waxy wheat line [18].

The aim of the current study was to identify orthologs of barley *Qsd1* in hexaploid wheat. Barley and wheat have closely related genomes, with similarities in the sequences of genes and repeated elements. We also aimed to identify dormancy loci to develop ways to prevent pre-harvest sprouting, since the dormancy period of wild barley harboring *Qsd1* is more than 5 weeks, whereas no seed dormancy QTL having a comparable effect on dormancy period has been reported in hexaploid wheat. To identify wheat with high levels of seed dormancy, we obtained sequence information for *Qsd1* in the sub-genomes of wheat and compared these genes to those in barley and other wheat haplotypes.

## Methods

### Plant materials

Twenty seeds of hexaploid wheat cv. Chinese Spring (CS) were sown in a plastic box (650 × 225 mm) and grown in a greenhouse in Obihiro, Japan under natural conditions until heading. After heading, the plants were moved to a greenhouse under controlled temperature conditions (ca. 22 °C day/16 °C night) with natural light until maturity. Each 20 seeds of diploid wheat lines *T. monococcum* ssp. *monococcum* (syn. *T. monococcum*) KT3–5 and *T. monococcum* ssp. *aegilopoides* (syn. *T. boeoticum*) KT1–1 and recombinant inbred line RIL 56 (long dormancy) derived from a cross between KT3–5 and KT1–1 [15] were grown in an experimental greenhouse in Kurashiki, Japan. For DNA extraction, five seeds of hexaploid wheat cvs. Kitahonami, Haruyo Koi and Fielder were used. To obtain DNA and RNA samples, the seeds were germinated on Petri dishes and the seedlings were grown for 1 week in an incubator at 20 °C. Small pieces of first leaf tissue were collected for both DNA and RNA extraction, and seminal roots were collected for RNA extraction. To prepare RNA from CS embryos, developing embryos were collected every 5 days from 10 days after anthesis until maturity (35 days after anthesis).

### BAC clone selection and sequencing of cv. Chinese spring

The full-length cDNA sequence (RFL_Contig4246, GenBank: AK333743.1) of CS was identified in the Wheat Genetic Resources Database (http://shigen.nig.ac.jp/wheat/komugi/ests/blast.jsp), representing an ortholog of *Qsd1* from barley. A pair of primers was designated (Additional file 1: Table S1) based on the sequence of AK333743.1 using Primer3 (http://primer3.ut.ee) and was used for BAC library screening of CS. Distinct BAC clones were identified via fingerprinting of *Not*I-digested DNA. Each shotgun library of BAC clones was sequenced using a 3730xl Sequencer (Applied Biosystems). Reads were assembled into contigs using Phred/Phrap software (http://www.phrap.org/phredphrapconsed.html). PCR analysis was performed using DNA samples from a set of nullisomic-tetrasomic CS lines [19] for the homoeologous group 5 chromosomes to identify the origins of the sub-genomes.

### RNA extraction and qPCR of hexaploid wheat

Total RNA was extracted from developing embryos of CS using TRIzol reagent (Invitrogen, Carlsbad, CA). First-strand cDNA was synthesized with ReverTra Ace® qPCR RT Master Mix with gDNA Remover (Toyobo, Osaka, Japan). qRT-PCR analysis was performed using the LightCycler® 480 SYBR Green I Master (Roche) and a LightCycler® 480 Instrument II (Roche). Gene-specific primers were designated for *Qsd1* in each sub-genome as follows: forward, 5′-GCGAGGAGAAGATCAAGGAG-3′ and reverse, 5′-GCTTAATTTACAGGGTAGGGTAGAT-3′ for the A sub-genome; forward, 5′-GGCGAGGACAAGATCAAGGC-3′ and reverse, 5′-TTTACAAGGTAGTGAAGATCACACCT-3′ for the B sub-genome; and forward, 5′-TTCATGAACGAGTTCCGT-3′ and reverse, 5′-TTAATTTACAGGGTAGGGTAGTGA-3′ for the D sub-genome. The *Actin* gene was used as an internal control. A primer pair developed by Nakamura et al. [7], qRT-Actin-F: 5′-CTATGTTCCCGGGTATTGCT-3′ and qRT-Actin-R: 5′-AAGGGAGGCAAGAATCGAC-3′, was used to amplify *Actin*. Biological replicates of three independent RNA extractions per sample were performed.

### RACE-PCR and sequencing

To determine the full-length nucleotide sequences of *Qsd1* in the B and D sub-genomes, RACE-PCR was performed using RNA from CS with a SMARTer® RACE 5′/3′ Kit (Clontech, CA, USA). The 5′ end fragments of *Qsd1* in the B and D sub-genomes were amplified using genome-specific primers 5′-GATTACGCCAAGCTTTTTACAAGGTAGTGAAGATCACACCT-3′ and 5′-GATTACGCCAAGCTTAGGCTTAATTTACAGGGTAGGGTAGTG-3′, respectively. For the 3′ end, 5′-GATTACGCCAAGCTTGTCCCAAGAAGATGGCATTC-3′ and 5′-GATTACGCCAAGCTTATGTCGTACAACAAGACGGCGTC-3′, respectively, were utilized. The PCR products were cloned into the pRACE vector with an In-Fusion® HD Cloning Kit (Clontech) and sequenced on an ABI PRISM® 3130xl Genetic Analyzer (Applied Biosystems, Foster, CA, USA) according to the manufacturer’s protocol.

### Haplotype sequencing and alignment in hexaploid wheat

To sequence the three *Qsd1* genes in the A, B, and D sub-genomes of cvs. Haruyo Koi, Kitahonami, and Fielder, genome-specific primers were designed based on the alignment of three *Qsd1* sequences in CS BAC clones (Additional file 1: Table S1). Amplification of genome-specific regions was confirmed by gel electrophoresis using nullisomic-tetrasomic lines for the homoeologous group 5 chromosomes [19]. Amplicons were purified on a Labo Pass™ Gel using a DNA Purification Reagent Kit (Hokkaido System Science Ltd., Sapporo, Japan). Purified amplicons were sequenced using a Big Dye Terminator Kit v3.1 (Applied Biosystems, Foster, CA, USA) and analyzed using a 3730xl DNA Analyzer (Applied Biosystems, Foster, CA, USA).

### Diploid wheat sequencing

DNA samples were harvested for PCR from diploid wheat accessions *T. monococcum*, *T. boeoticum*, and RIL 56. Primer sets were developed based on primer information from Sato et al. [9] with some modifications (Additional file 1: Table S2). The amplicons were sequenced using a 3130xl Genetic Analyzer (Applied Biosystems).

Total RNA was extracted from maturing grains (embryos) at 4 weeks after flowering using the NucleoSpin® RNA Plant system (MACHEREY-NAGEL, Düren, Germany). First-strand cDNA was synthesized with ReverTra Ace® qPCR RT Master Mix with gDNA Remover (Toyobo, Osaka, Japan). The qRT-PCR analysis was performed using THUNDERBIRD® SYBR® qPCR Mix (Toyobo) on a StepOnePlus™ Real Time PCR System (Applied Biosystems, Foster, CA, USA) according to the manufacturer’s protocol. A primer pair developed by Nakamura et al. [7], qRT-Actin-F: 5′-CTATGTTCCCGGGTATTGCT-3′ and qRT-Actin-R: 5′-AAGGGAGGCAAGAATCGAC-3′, was used to amplify *Actin*. Biological replicates of three independent RNA extractions per sample were performed. The cDNA sequences of these three accessions were obtained using primer sets based on the barley Qsd1 amino acid sequence (Additional file 1: Table S2).

## Results

### *BAC sequencing of* Qsd1 *homeologs in cv. Chinese Spring*

We used a pair of primers (Contig4246-1_L and Contig4246-1_R) based on the full-length cDNA sequence homologous to barley *Qsd1* (RFL_Contig4246, GenBank: AK333743.1) to select BAC clones harboring *Qsd1* homeologs. Two distinct BAC clones (WCS0334P24 and WCS0897G21) were identified based on fingerprinting of *Not*I-digested DNA. We mapped the primer sequences onto contig sequences to confirm that the target sequences were included in the BAC clones. We designed primer pair 0334P24-L1–1 and 0334P24-R1–2 to distinguish between amplicons from WCS0334P24 and WCS0897G21 based on the presence of an indel at intron 14 (Additional file 1: Figure S1). Using DNA samples from a set of nullisomic-tetrasomic (NT) lines [19] for homoeologous group 5 chromosomes, we obtained three amplicons by PCR, two of which corresponded to WCS0334P24 and WCS0897G21, respectively. The sequences from WCS0334P24 (DDBJ no. LC209615) and WCS0897G21 (LC209617) were derived from chromosomes 5A and 5D, respectively, and the additional amplicon was derived from chromosome 5B (Additional file 1: Figure S2). To identify positive BAC clones from chromosome 5B, we conducted a second screening of the BAC library using primer pair 0334P24-L1–1 and 0334P24-R1–2 and sequenced one BAC clone, WCS1890O23, derived from chromosome 5B (LC209616).

### *Structures of* Qsd1 *in wheat*

**Fig. 1:**
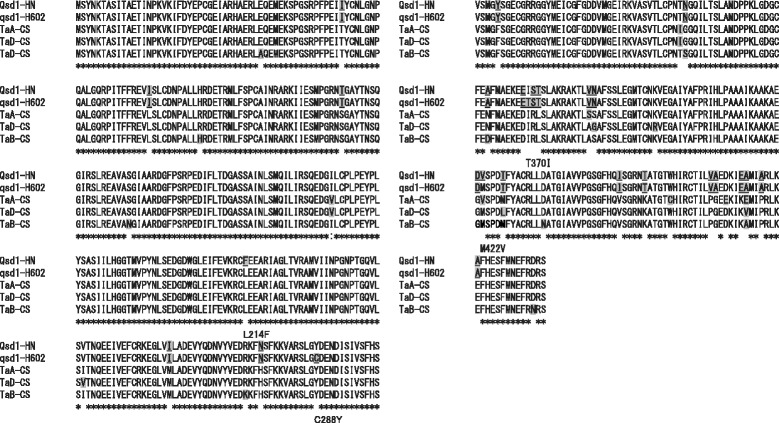
Comparison of amino acid sequences of Qsd1 among barley cv. Haruna Nijo (Qsd1-HN; short dormancy), wild accession H602 (qsd1-H602: long dormancy), and the three sub-genomes of wheat cv. Chinese Spring (TaA-CS, TaB-CS, and TaD-CS). Amino acids highlighted in *gray* show minor substitutions among haplotypes. Barley-specific amino acid substitutions are *underlined*. The amino acid numbers and substituted amino acids *beneath the alignment* show barley-specific substitutions between Haruna Nijo and H602

Since only one homologous full-length *Qsd1* sequence was available from the Wheat Genetic Resources Database, which was identified as belonging to the wheat A sub-genome by BAC sequencing, we obtained transcript sequences from the other sub-genomes by 5′ and 3′ RACE using RNA samples isolated from embryos (Fig. 1). The amino acid sequences deduced from the *Qsd1* sequences in the B and D sub-genomes (LC209618 and LC209619) completely matched the deduced amino acid sequences (UniProt ID W5FER5 and W5FWW5, respectively) from transcripts in the genome assembly TGACv1 in Ensemble Plants (http://plants.ensembl.org/Triticum_aestivum/Info/Index). We aligned the BAC clone sequences, full-length cDNA sequence (AK333743.1), and transcript sequences (LC209618 and LC209619) using CLUSTALW (http://www.genome.jp/tools/clustalw/) to estimate exon/intron structures and start/stop codons (Additional file 2: Figure S3a). There were no differences in exon/intron structures among the three sub-genomes (Additional file 2: Figure S3a). All transcript sequences of *Qsd1* were aligned to estimate amino acid substitutions among the wheat and barley genomes (Fig. 1). We detected 13 amino acid substitutions among the three amino acid sub-genome sequences in CS. Numerous sequence polymorphisms are also present among the intron regions of the sub-genomes, as shown in Additional file 2: Figure S3a.

We sequenced the locus (LC209620 and LC209621) and mRNA (LC209622 and LC209623) of *Qsd1* orthologs in diploid wheat lines *T. monococcum* KT3–5 (Tm) and *T. boeoticum* KT1–1 (Tb), respectively. We also sequenced the locus of RIL 56, and found that it was identical to that of Tm. We aligned the genomic and mRNA sequences of two A genome diploid wheat accessions and the CS A sub-genome; the exons, start/stop codons, and polymorphic nucleotides are shown in Additional file 2: Figure S3b. Very high sequence similarity was observed among the three haplotypes, especially between Tm (*TmA*) and Tb (*TbA*)*.*

### *Amino acid sequence alignment of* Qsd1 *orthologs*

**Fig. 2:**
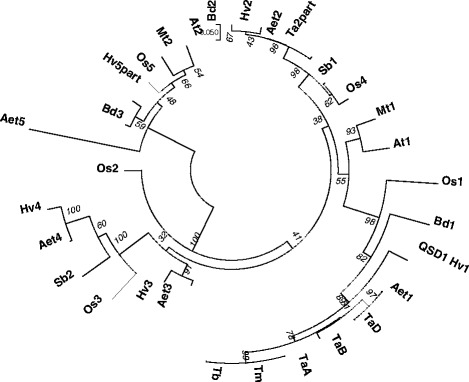
Multiple alignment of QSD1 amino acid sequences (Hv1: Haruna Nijo). The listed sequences are from accessions showing similarity to QSD1 (E-value < E-145) by Blastp analysis of NCBI nr. Species and homologs (with accession numbers) are as follows: *Hordeum vulgare*: Hv1 (BAK04026.1), Hv2 (BAK07780.1), Hv3 (P52894.1), Hv4 (BAK05632.1), Hv5part (BAJ90574.1); *Triticum aestivum*: TaA (AK333743.1), TaB (LC209618), TaD (LC209619), Ta2part (CAE54279.1); *T. monococcum*: Tm (LC209623); *T. boeoticum*: Tb (LC209622); *Aegilops tauschii*: Aet1 (EMT25616.1), Aet2 (EMT05433.1), Aet3 (EMT23015.1), Aet4 (EMT08497.1), Aet5EMT29455.1); *Oryza sativa*: Os1 (NP_001063248.1), Os2 (NP_001064504.1) Os3 (NP_001064505.2), Os4 (NP_001060284.1), Os5 (NP_001058716.1); *Sorghum bicolor*: Sb1 (XP_002463187.1), Sb2 (XP_002467302.1); *Brachypodium distachyon*: Bd1 (XP_003578159.1), Bd2 (XP_010235387.1), Bd3 (XP_003557680.1); *Arabidopsis thaliana*: At1 (NP_173173.3), At2 (AAK68842.1); *Medicago truncatula*: Mt1 (XP_003627448.1), Mt2 (XP_003613139.1). *Scale bar* indicates Poisson Correction distance. *Numerals* show test values for 1000 bootstrap replications

We identified amino acid sequences from accessions showing similarity to QSD1 (protein from short dormancy barley cv. Haruna Nijo) by Blastp analysis (E-value < E-145) of sequences in NCBI nr. The sequences of *Hordeum vulgare*, *Triticum aestivum*, *T. monococcum*, *T. boeoticum*, *Aegilops tauschii*, *Oryza sativa* (rice), *Sorghum bicolor* (sorghum), *Brachypodium distachyon*, *Arabidopsis thaliana*, and *Medicago truncatula* were aligned to estimate the evolutionary distances among orthologs (Fig. 2). The distances between wheat and barley QSD1 were the shortest among the plant amino acid comparisons. Both barley and rice contain five members of the *Qsd1* gene family [9]. The same Blastp search of *T. aestivum* (Ta) resulted in the identification of only one partial sequence (Ta2part); however, every member of this gene family was available for *Ae. tauschii*, which is a donor of the D genome in hexaploid wheat (Fig. 2). The amino acid sequence of CS-TaB was the most different among the three sub-genomes of Ta, but it was closer to Tm, Tb, and barley QSD1 (Fig. 2).

### *Expression profiles of wheat* Qsd1

**Fig. 3:**
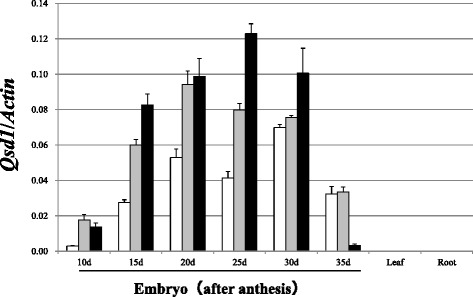
Relative expression levels of wheat *Qsd1*/*Actin* (Additional file 1: Figure S5) in the three sub-genomes of cv. Chinese Spring (*open bar*:*TaA*, *grey bar*:*TaB,* and *black bar*:*TaD*) in different organs and at different growth stages. Error bars represent standard error, *n* = 3

We investigated the expression profiles of all three orthologs of *Qsd1* in the A, B, and D sub-genomes of CS (*TaA*, *TaB*, and *TaD*, respectively) via qRT-PCR using homeolog-specific primer pairs designed based on exon 15 and the 3’UTRs (Additional file 1: Table S1). Like *Qsd1/qsd1* in barley, transcripts for all homeologs were detected in developing embryos, whereas very low levels of expression were found in leaves and roots (Fig. 3, Additional file 1: Figure S4). For all homeologous orthologs of *Qsd1*, the expression levels increased until 20 to 30 days after anthesis and decreased toward maturation. *TaA* was expressed at relatively low levels compared to *TaB* and *TaD* during the maturation period. *TaD* was highly expressed at most time points but was expressed at very low levels at the last time point (during maturity; day 35).

We measured *Qsd1* expression in diploid wheat (*Tm, Tb*, and RIL56) on day 28, finding no difference in expression among accessions (Additional file 1: Figures S5 and S6).

### *Sequence comparison of* Qsd1 *among elite wheat haplotypes*

**Fig. 4:**
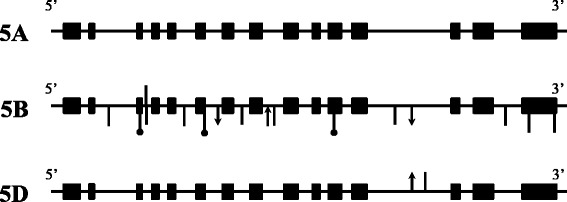
Comparison of sequences of loci in four haplotypes of hexaploid wheat cultivars. *Square*: exon; *line*: synonymous SNP; *line with rounded end*: asynonymous SNP; *upward arrow*: insertion; *downward arrow*: deletion. *Vertical lines beneath* the chromosome indicate polymorphisms between Kitahonami and Chinese Spring (CS). The *vertical line above* the chromosome indicates a polymorphism between Haruyo Koi and CS. Fielder and CS did not show any differences among the three sub-genomes

We determined the sequences of *Qsd1* loci for the A, B, and D sub-genomes of Japanese winter cultivar Kitahonami, Japanese spring wheat cultivar Haruyo Koi, and Canadian spring wheat cultivar Fielder (DDBJ no. LC209824 - LC209832) and compared them with those of CS (Fig. 4). There were no sequence polymorphisms in the A sub-genome, whereas there were two nucleotide sequence differences in *TaD* between CS and Haruyo Koi. Numerous sequence polymorphisms were identified in the B sub-genome derived from the difference between CS and Kitahonami (Fig. 4). Compared to the CS sequences, three non-synonymous substitutions were found in exons 3, 6, and 11 of *TaB* in Kitahonami, whereas no polymorphisms were found in exons of the A and D sub-genomes. Finally, no sequence polymorphisms were found between CS and Fielder, indicating that these haplotypes share a closely related phylogeny.

We aligned the amino acid sequences of the three sub-genomes of CS, diploid wheat (*Tm* and *Tb*), and the B sub-genome of Kitahonami with the sequences of both Haruna Nijo (Qsd1: short dormancy) and H602 (qsd1: long dormancy) in barley (Additional file 1: Figure S7). We detected four amino acid substitutions between Haruna Nijo and H602, but no amino acid sequence polymorphisms were found in wheat at the critical L214F substitution, which is responsible for the difference in seed dormancy period between Haruna Nijo and H602. While polymorphisms were detected among wheat sub-genomes at the M422V substitution, no polymorphisms were found between CS and Kitahonami at this position.

## Discussion

### *Sequence similarity of* Qsd1 *among wheat and barley accessions*

The AlaAT gene family has five members, as determined based on a similarity search for barley *Qsd1* by Blastp in NCBI nr. These orthologous sequences are present in rice, *Aegilops tauschii*, and barley, although only a partial sequence is currently available for barley (Hv5part) (Fig. 2). The availability of amino acid sequences depends on the progress in annotating in each species. Since the complete genome sequence of hexaploid wheat has not yet published, the annotation information for hexaploid wheat is not fully available in the public database. *Brachypodium distachyon* (Bd) is thought to be more closely related to Triticeae species (*Hordeum*, *Triticum*, *Secale*, and *Aegilops* in Fig. 2) than to rice, but only three AlaAT gene family members have been identified in Bd. The sequences of *Qsd1* gene family members in *Ae. tauschii* showed a closer relationship to barley than to rice in every comparison of the five members of this family. Although only one partial amino acid sequence (Ta2part) is available for the *Qsd1* gene family in hexaploid wheat (as of March 2, 2017), all five members might be identified for each sub-genome after the release of the genome sequence, or perhaps a few might have been lost during the polyploidization of hexaploid wheat.

All three wheat *Qsd1* orthologs from CS share close sequence similarity with barley *Qsd1.* There are 13 amino acid substitutions among the CS sub-genomes and 20 additional substitutions between barley cv. Haruna Nijo and CS (Fig. 1). Sato et al. [9] proposed that the change from a phenylalanine residue in cv. Haruna Nijo (short dormancy) to a leucine residue at amino acid position 214 in wild barley H602 (long dormancy) is a major mutation that occurred during the transition from long-dormancy to short-dormancy barley during the selection of short-dormancy barley for malting purposes. While in every CS sub-genome, the amino acid at position 214 is leucine (Fig. 1), the results suggest that the 214F mutation is barley-specific and is not common among major plant species and wheat sub-genomes.

The mutation underlying the transition from long dormancy to short dormancy is dominant and gain of function, as Sato et al. [9] demonstrated in an RNAi experiment to obtain long dormancy transgenic plants in barley cv. Golden Promise (Qsd1: short dormancy). Dominant short dormancy was also found in another dormancy QTL, *Qsd2*, in barley [8], whose homolog is present in hexaploid wheat [14]. Takeda and Hori [20] evaluated 4365 cultivated barley and 177 wild barley (*H. vulgare* ssp. *spontaneum*) accessions from Okayama University and found that most showed long dormancy. Suzuki [21] evaluated seed dormancy in wild and cultivated *Triticum* and *Aegilops* species and found that wild wheat accessions had longer dormancy periods. We propose that the short dormancy mutation occurred during the evolutionary process, particularly after domestication, and that shorter dormancy genotypes were selected by humans. While there are many amino acid substitutions among the wheat sub-genomes, the sequence differences among cultivars are smaller than those among sub-genomes in a cultivar, and in A genome diploids. We only detected differences in the B sub-genome of winter wheat cv. Kitahonami; these differences may be investigated in the future for their role in longer dormancy.

### *Differentiation of* Qsd1 *orthologs*

We demonstrated that the *Qsd1* orthologs were expressed in wheat embryos but not in leaves or roots (Fig. 3). These results are in agreement with the expression profiles and organ specificity of *Qsd1* in barley, as well as the rice *Qsd1* ortholog [9]. The results also support the homeology of barley and wheat *Qsd1* sequences observed in the present study. While there are some differences in the expression profiles of the three *Qsd1* orthologs in the wheat sub-genomes, all of these genes are expressed and may be functional throughout maturation (Fig. 3). The amino acid substitutions among the three sub-genome sequences in CS (Fig. 1) suggest that each protein derived from a sub-genome has a different structure and that TaB is the most distinct of the sub-genomes. However, based on the evolutionary relationship among related species, TaB may share a closer origin with other species, especially barley, and TaA and TaD are more distantly related to other species. The expression of this gene did not significantly differ among diploid wheat accessions at maturity (Additional file 1: Figure S5).

### *Application of wheat* Qsd1 *homeologs*

We expanded our sequence comparison to three other hexaploid wheat cultivars with available dormancy information. The alignment of four hexaploid wheat cultivars indicated that the amino acid sequences of three spring cultivars, CS, Haruyo Koi, and Fielder, are exactly the same in each sub-genome. Only Kitahonami has three amino acid substitutions at TaB from spring wheat cultivars. Fielder is in a Canadian wheat class of Canada Western Soft White Spring with white-seed-coat which is reported as a class with low level of seed dormancy [22]. Chono et al. [23] tested the germination indices of 324 wheat cultivars to evaluate pre-harvest sprouting (0 [tolerant] to 100 [sensitive]), finding that Kitahonami had indices of 11.4 (2007) and 11.9 (2008) and that CS had indices of 83.8 (2007) and 97.6 (2008). Yanagisawa et al. [24] also scored the level of pre-harvest sprouting in Kitahonami as moderately tolerant. Onishi (unpublished data) scored Haruyo Koi and CS for 2 years, finding no differences in seed dormancy. Germination rates (20 °C, 10 days) of CS and Haruyo Koi were 63.6% (*n* = 10) and 72.1% (*n* = 3) in 2013 (*p* = 0.62, t-test), 75.0% (*n* = 6) and 72.5% (*n* = 3) in 2014 (*p* = 0.61, t-test), respectively. As Cao et al. [25] mapped a seed dormancy QTL derived from CS, which has a certain level of dormancy, the pre-harvest sprouting score of CS is assumed to be moderate. Since some substitutions exist in the B sub-genomes between CS and Kitahonami, these substitutions should be evaluated for their roles in seed dormancy in the future.

## Conclusions

The sequence information obtained in this study and the PCR primers used to identify each sub-genome may contribute to resequencing efforts for other wheat haplotypes with known levels of seed dormancy. On the other hand, no extremely long dormancy effect (such as that observed for the *qsd1* allele in wild barley) has been found in wheat. The results of sequence comparison among barley and wheat genomes suggest that the 214F mutation is barley-specific and is not common among major plant species and wheat sub-genomes. We only detected differences in the B sub-genome of winter wheat cv. Kitahonami; these differences may be investigated in the future for their role in longer dormancy. Since extremely long dormancy levels are found among wild wheat species [21], perhaps their *Qsd1* orthologs could be resequenced to identify allelic mutations as an alternative strategy for mining longer dormancy sources in cultivated wheat.

## Additional files

Additional file 1: Table S1. Primers used for Chinese Spring BAC library selection. **Table S2.** Primer information for polymorphism detection in the *Qsd1* region. The position is based on the numbers from the 3′ end of a barley cv. Haruna Nijo BAC clone [9]. **Figure S1 a.** Primer positions (arrows) for clone selection from the Chinese Spring (CS) BAC library. WCS0334P24 has two insertions in intron 14 as indicated by orange wedges. **b** Amplification of DNA samples from a set of nullisomic-tetrasomic (NT) lines [19] of CS for the homeologous group 5 chromosomes. **Figure S2.** Primer positions (arrows) for sequencing and amplification of DNA samples from a set of nullisomic-tetrasomic (NT) lines (Sears [19]) of CS for homeologous group 5 chromosomes. Numbers show accessions: 1. CS; 2. N5AT5B; 3. N5AT5D; 4. N5BT5A; 5. N5BT5D; 6. N5DT5B; 7. Kitahonami; 8. Haruyo Koi. Red line with marker name is a position for primer amplification corresponding to Table S1. **Figure S4.** The plot of the log copy number versus threshold cycle (Ct) and the regression line for the expression of *Qsd1* from Chinese Spring shown in Fig. 2. **Figure S5.** Expression levels of *Qsd1* relative to *Actin* in embryos at 28 d after flowering. Error bars represent standard error, *n* = 3. **Figure S6.** The plot of the log copy number versus threshold cycle (Ct) and the regression line for the expression of *Qsd1* in diploid wheat accessions shown in Figure S5. **Figure S7.** Comparison of Qsd1 orthologous amino acid sequences in wheat and barley. Asterisks indicate no substitution among the materials. (PPTX 980 kb)

Additional file 2: Figure S3 a. Nucleotide sequences of *Qsd1* orthologous loci in the sub-genomes of cv. Chinese Spring (TaA-CS, TaB-CS, and TaD-CS). The cDNA sequences are underlined. Start and stop codons are shown in bold. Introns are shown in standard font. **b** Nucleotide sequences of *Qsd1* orthologs in the A sub-genomes of cv. Chinese Spring (TaA-CS), *Triticum boeoticum* (TbA), and *T. monococcum* (TmA). The cDNA sequences are underlined. Polymorphisms between the A sub-genome vs. diploid wheat and within diploid wheat accessions are highlighted in green and purple, respectively. Start and stop codons are shown in bold. The primer positions used for sequencing are highlighted in gray and marker names are shown in parentheses. (ZIP 43 kb)

## Abbreviations

BAC: Bacterial artificial chromosome
qRT-PCR: Quantitative reverse transcription polymerase chain reaction
QTL: Quantitative trait locus
RACE: Rapid amplification of cDNA ends
RNAi: RNA interference
SNP: Single nucleotide polymorphism
