# Erratum to: Sequence differences in the seed dormancy gene *Qsd1* among various wheat genomes

**DOI:** 10.1186/s12864-017-4046-2

**Published:** 2017-08-29

**Authors:** Kazumitsu Onishi, Miki Yamane, Nami Yamaji, Mayumi Tokui, Hiroyuki Kanamori, Jianzhong Wu, Takao Komatsuda, Kazuhiro Sato

**Affiliations:** 1 Obihiro University of Agriculture and Veterinary Medicine, Obihiro, 080-8555 Japan; 2 Institute of Plant Science and Resources, Okayama University, Kurashiki, 710-0046 Japan; 3 Institute of Crop Science, National Agriculture and Food Research Organization, Tsukuba, 305-8634 Japan

## Erratum

After the publication of this article [1] the authors noted that Fig. 2 was incorrect and a number of species abbreviations were not in their intended positions, such as At2 and Bd2.

A correct version of Fig. 2 is included with this Erratum.

The original article has been corrected.

**Fig. 2:**
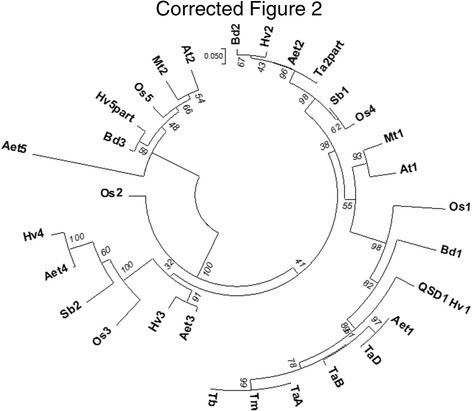
Multiple alignment of QSD1 amino acid sequences (Hv1: Haruna Nijo). The listed sequences are from accessions showing similarity to QSD1 (E-value < E-145) by Blastp analysis of NCBI nr. Species and homologs (with accession numbers) are as follows: *Hordeum vulgare*: Hv1 (BAK04026.1), Hv2 (BAK07780.1), Hv3 (P52894.1), Hv4 (BAK05632.1), Hv5part (BAJ90574.1); *Triticum aestivum*: TaA (AK333743.1), TaB (LC209618), TaD (LC209619), Ta2part (CAE54279.1); *T. monococcum*: Tm (LC209623); *T. boeoticum*: Tb (LC209622); *Aegilops tauschii*: Aet1 (EMT25616.1), Aet2 (EMT05433.1), Aet3 (EMT23015.1), Aet4 (EMT08497.1), Aet5EMT29455.1); *Oryza sativa*: Os1 (NP_001063248.1), Os2 (NP_001064504.1) Os3 (NP_001064505.2), Os4 (NP_001060284.1), Os5 (NP_001058716.1); *Sorghum bicolor*: Sb1 (XP_002463187.1), Sb2 (XP_002467302.1); *Brachypodium distachyon*: Bd1 (XP_003578159.1), Bd2 (XP_010235387.1), Bd3 (XP_003557680.1); *Arabidopsis thaliana*: At1 (NP_173173.3), At2 (AAK68842.1); *Medicago truncatula*: Mt1 (XP_003627448.1), Mt2 (XP_003613139.1). Scale bar indicates Poisson Correction distance. Numerals show test values for 1000 bootstrap replications
