# Supplementary material for: Sequence differences in the seed dormancy gene Qsd1 among various wheat genomes
Source: BMC Genomics. 2017 Jun 29;18:497. doi: 10.1186/s12864-017-3880-6 (PMC5492916; doi:10.1186/s12864-017-3880-6)
Supplement: Supplementary file 1 — Primers used for Chinese Spring BAC library selection. Table S2. Primer information for polymorphism detection in the Qsd1 region. The position is based on the numbers from the 3′ end of a barley cv. Haruna Nijo BAC clone [9]. Figure S1 a. Primer positions (arrows) for clone selection from the Chinese Spring (CS) BAC library. WCS0334P24 has two insertions in intron 14 as indicated by orange wedges. b Amplification of DNA samples from a set of nullisomic-tetrasomic (NT) lines [19] of CS for the homeologous group 5 chromosomes. Figure S2. Primer positions (arrows) for sequencing and amplification of DNA samples from a set of nullisomic-tetrasomic (NT) lines (Sears [19]) of CS for homeologous group 5 chromosomes. Numbers show accessions: 1. CS; 2. N5AT5B; 3. N5AT5D; 4. N5BT5A; 5. N5BT5D; 6. N5DT5B; 7. Kitahonami; 8. Haruyo Koi. Red line with marker name is a position for primer amplification corresponding to Table S1. Figure S4. The plot of the log copy number versus threshold cycle (Ct) and the regression line for the expression of Qsd1 from Chinese Spring shown in Fig. 2. Figure S5. Expression levels of Qsd1 relative to Actin in embryos at 28 d after flowering. Error bars represent standard error, n = 3. Figure S6. The plot of the log copy number versus threshold cycle (Ct) and the regression line for the expression of Qsd1 in diploid wheat accessions shown in Figure S5. Figure S7. Comparison of Qsd1 orthologous amino acid sequences in wheat and barley. Asterisks indicate no substitution among the materials. (PPTX 980 kb) [file 12864_2017_3880_MOESM1_ESM.pptx]

## Slide 1
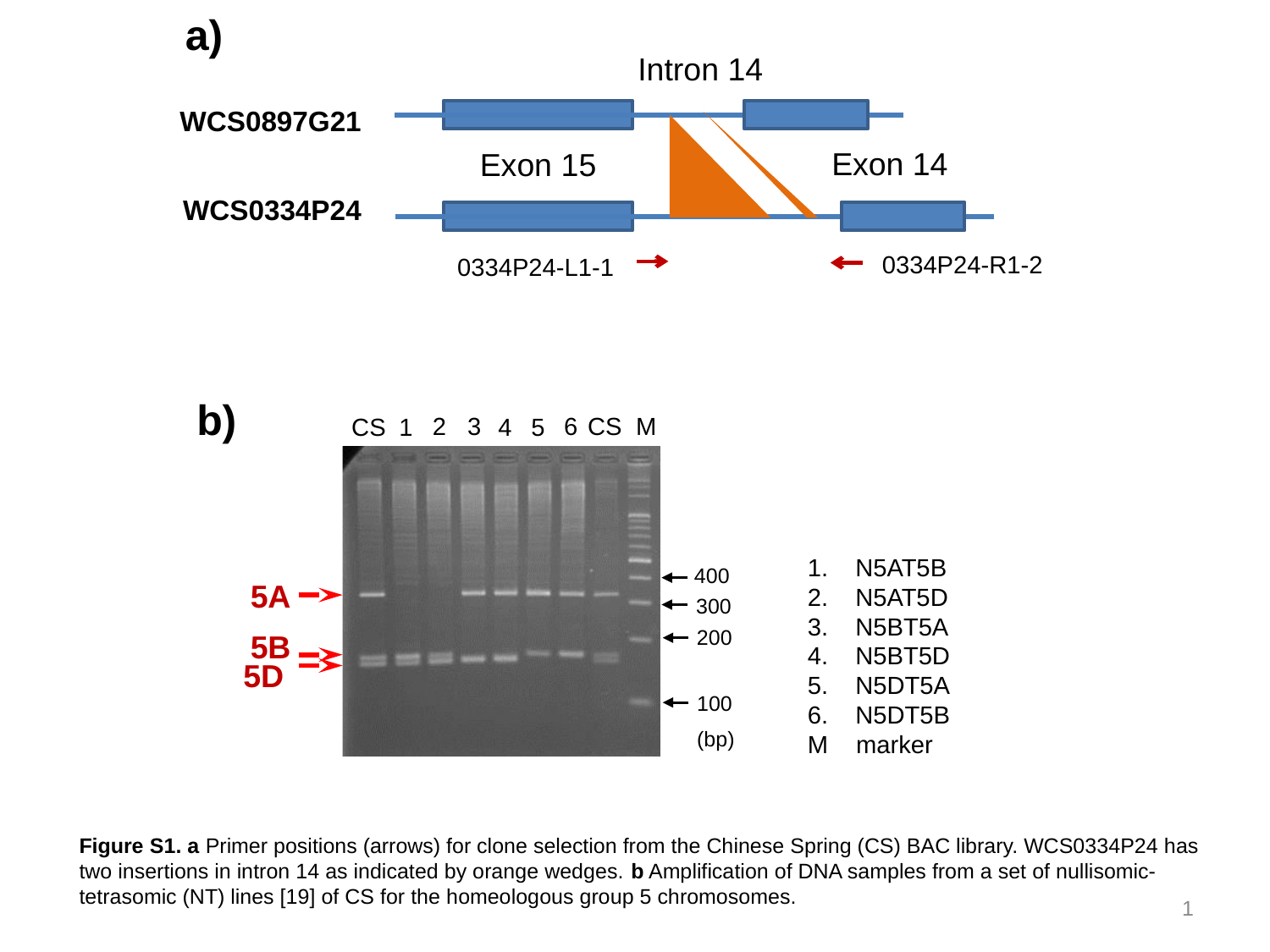

a)
Intron 14
WCS0897G21
Exon 14
Exon 15
WCS0334P24
0334P24-R1-2
0334P24-L1-1
b)
3
2
CS M
6
CS
1
5
4
400
5A
300
200
5B
5D
100
(bp)
N5AT5B
N5AT5D
N5BT5A
N5BT5D
N5DT5A
N5DT5B
M marker
Figure S1. a Primer positions (arrows) for clone selection from the Chinese Spring (CS) BAC library. WCS0334P24 has two insertions in intron 14 as indicated by orange wedges. b Amplification of DNA samples from a set of nullisomic-tetrasomic (NT) lines [19] of CS for the homeologous group 5 chromosomes.
1

## Slide 2
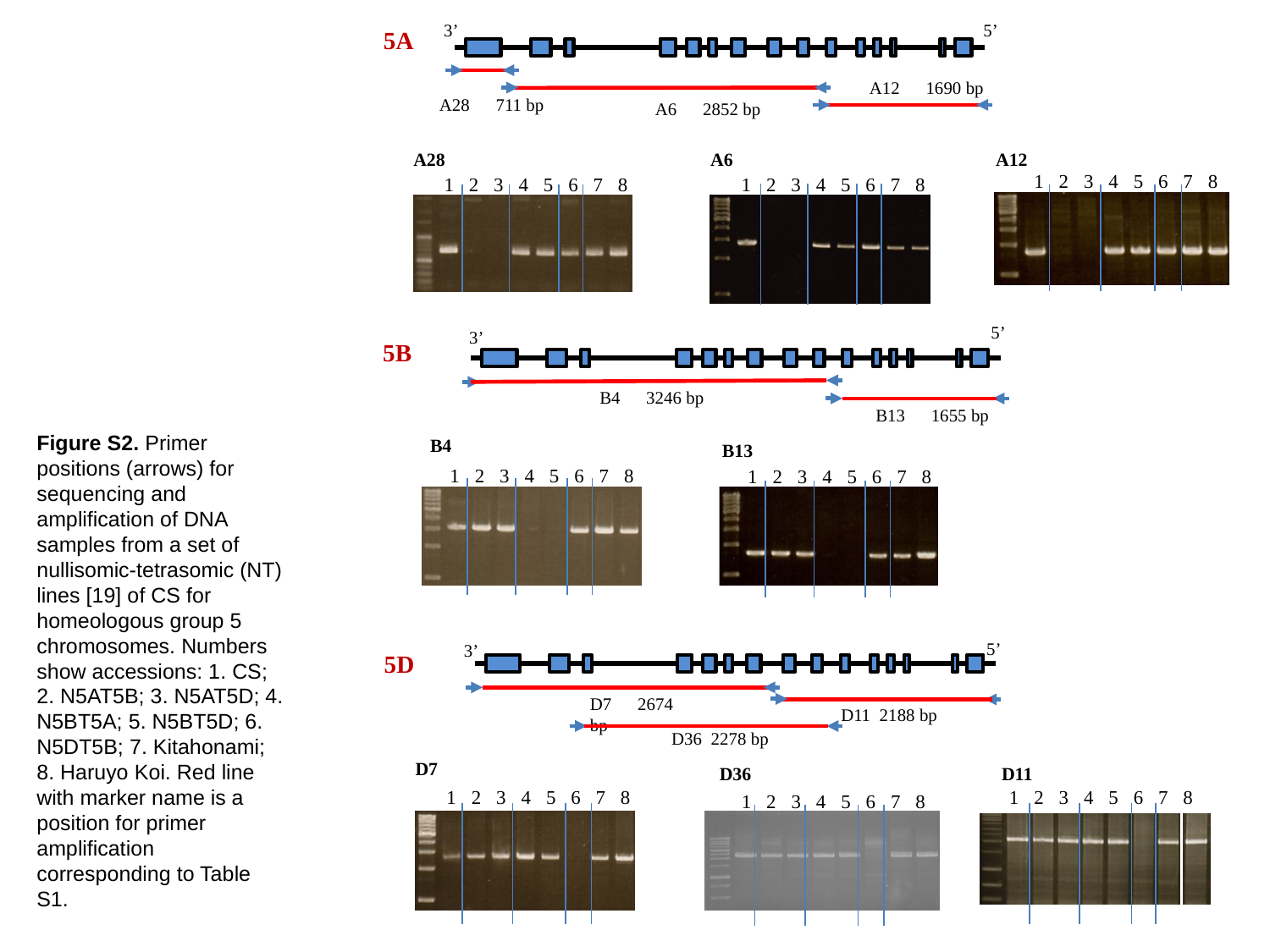

3’
5’
5A
A12　1690 bp
A28　711 bp
A6　2852 bp
A28
A6
A12
1
2
3
4
5
6
7
8
1
2
3
4
5
6
7
8
1
2
3
4
5
6
7
8
5’
3’
B4　3246 bp
B13　1655 bp
5B
Figure S2. Primer positions (arrows) for sequencing and amplification of DNA samples from a set of nullisomic-tetrasomic (NT) lines [19] of CS for homeologous group 5 chromosomes. Numbers show accessions: 1. CS; 2. N5AT5B; 3. N5AT5D; 4. N5BT5A; 5. N5BT5D; 6. N5DT5B; 7. Kitahonami; 8. Haruyo Koi. Red line with marker name is a position for primer amplification corresponding to Table S1.
B4
B13
1
2
3
4
5
6
7
8
1
2
3
4
5
6
7
8
5’
3’
D7　2674 bp
D11 2188 bp
D36 2278 bp
5D
D7
D36
D11
1
2
3
4
5
6
7
8
1
2
3
4
5
6
7
8
1
2
3
4
5
6
7
8

## Slide 3
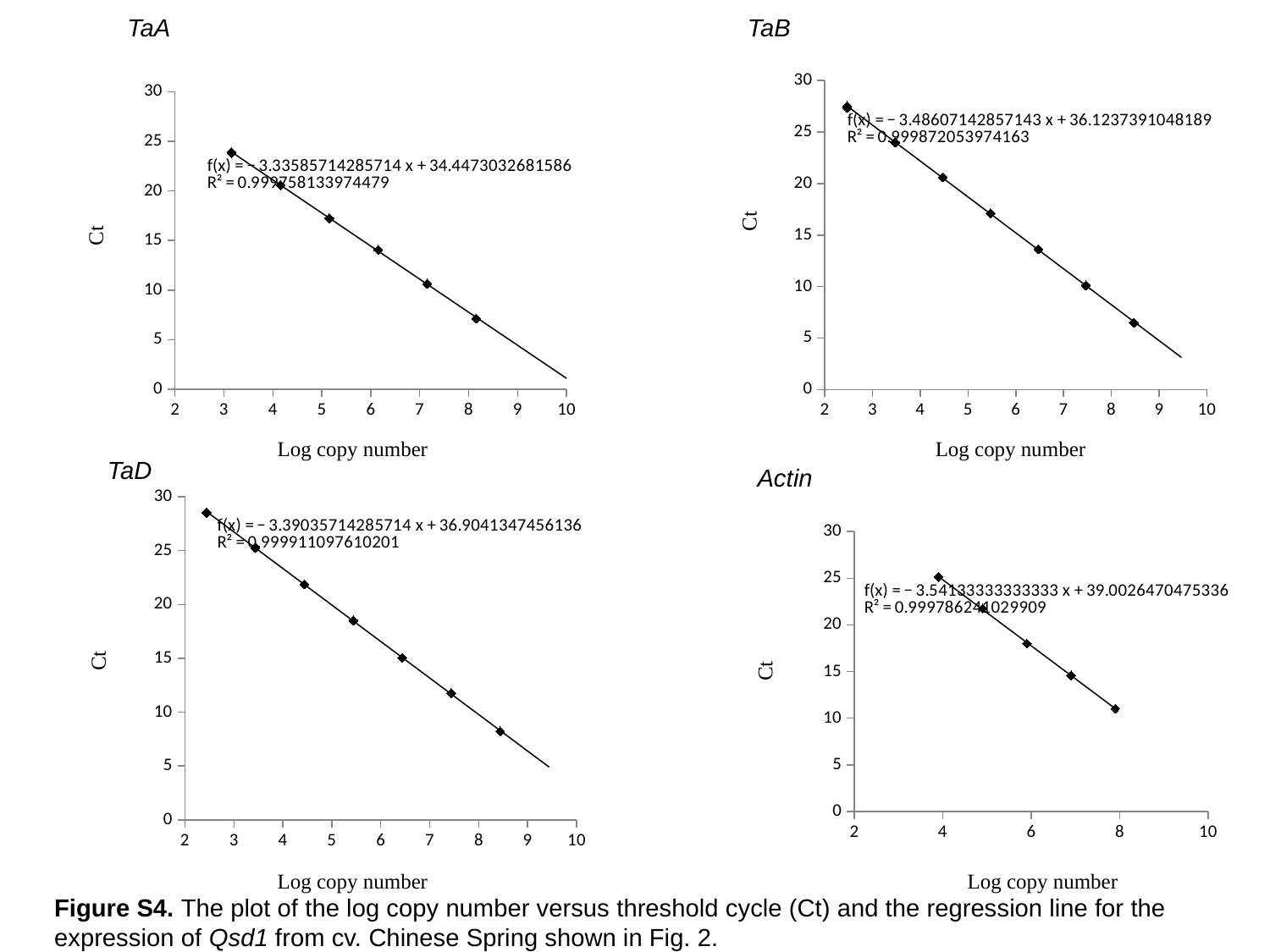

TaA
TaB
### Chart
| Category | |
|---|---|
### Chart
| Category | |
|---|---|Ct
Ct
Log copy number
Log copy number
TaD
Actin
### Chart
| Category | |
|---|---|
### Chart
| Category | |
|---|---|Ct
Ct
Log copy number
Log copy number
Figure S4. The plot of the log copy number versus threshold cycle (Ct) and the regression line for the expression of Qsd1 from cv. Chinese Spring shown in Fig. 2.

## Slide 4
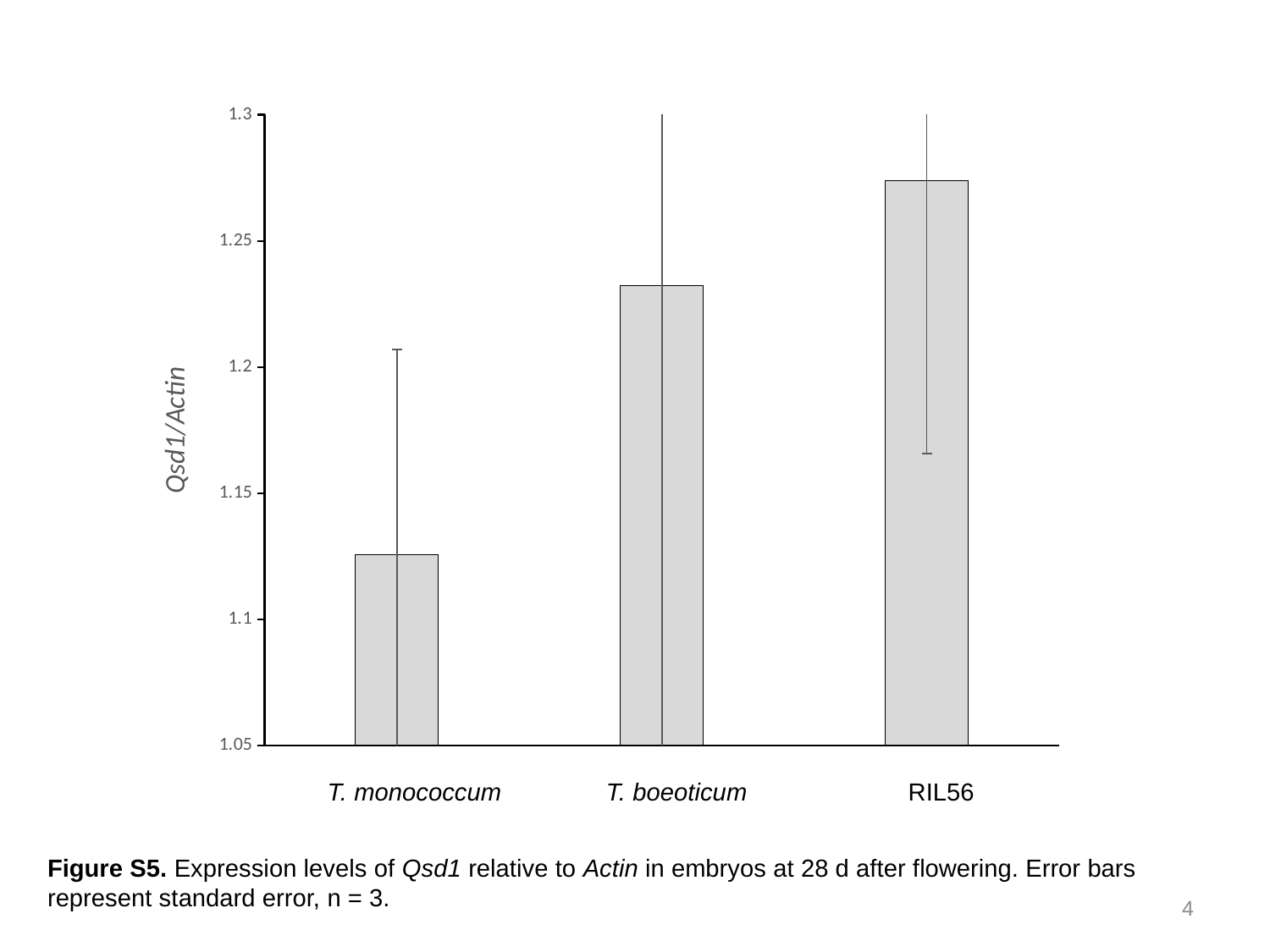

### Chart
| Category | TaQsd1/TaActin |
|---|---|
| T. monococum | 1.1254643489047131 |
| T. boeoticum | 1.2320848603630488 |
| RIL56 | 1.2739212551848242 |T. monococcum T. boeoticum RIL56
Figure S5. Expression levels of Qsd1 relative to Actin in embryos at 28 d after flowering. Error bars represent standard error, n = 3.
4

## Slide 5
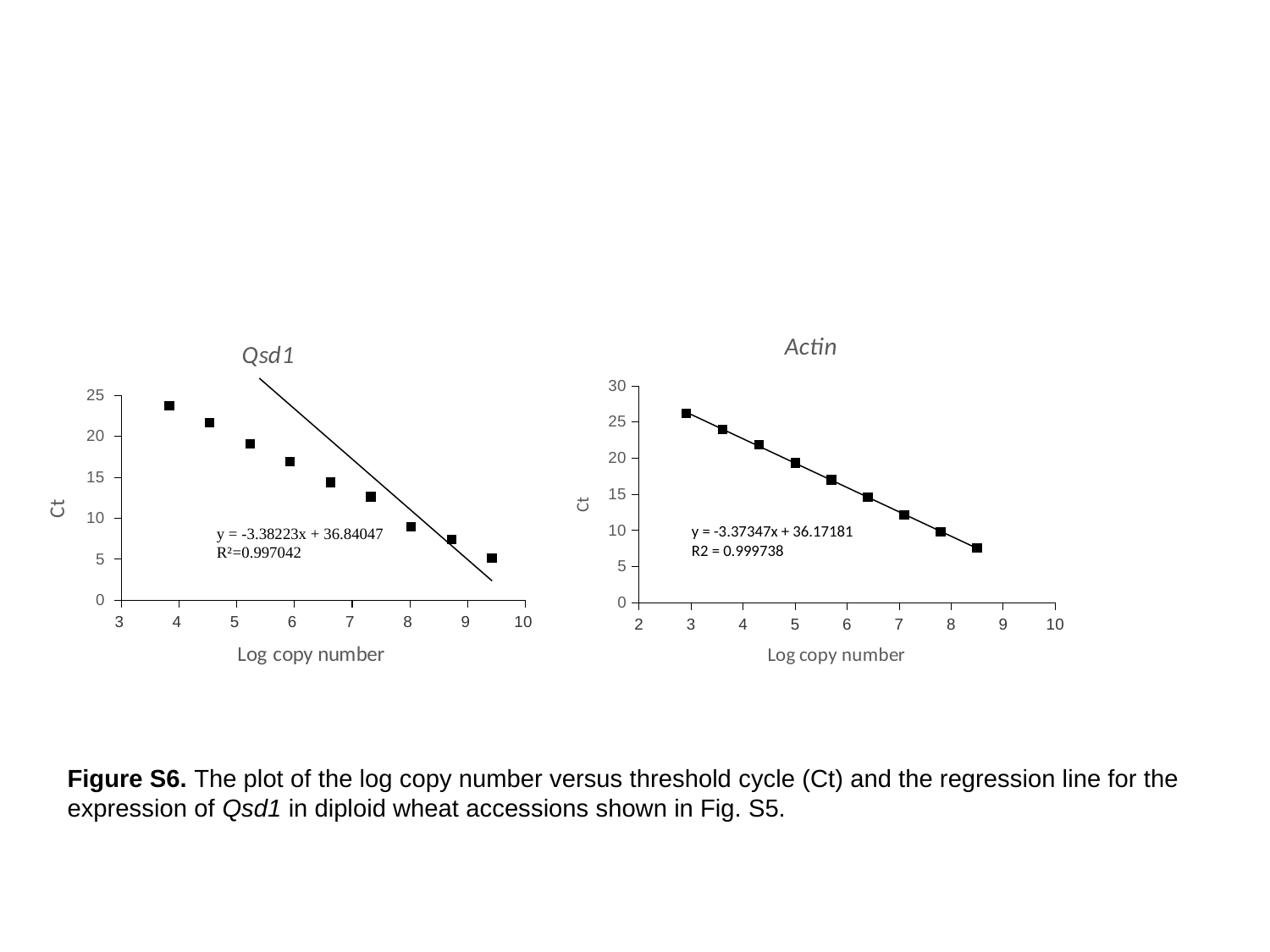

### Chart: Actin
| Category | Cт Mean |
|---|---|
### Chart: Qsd1
| Category | |
|---|---|y = -3.37347x + 36.17181
R2 = 0.999738
Figure S6. The plot of the log copy number versus threshold cycle (Ct) and the regression line for the expression of Qsd1 in diploid wheat accessions shown in Fig. S5.

## Slide 6
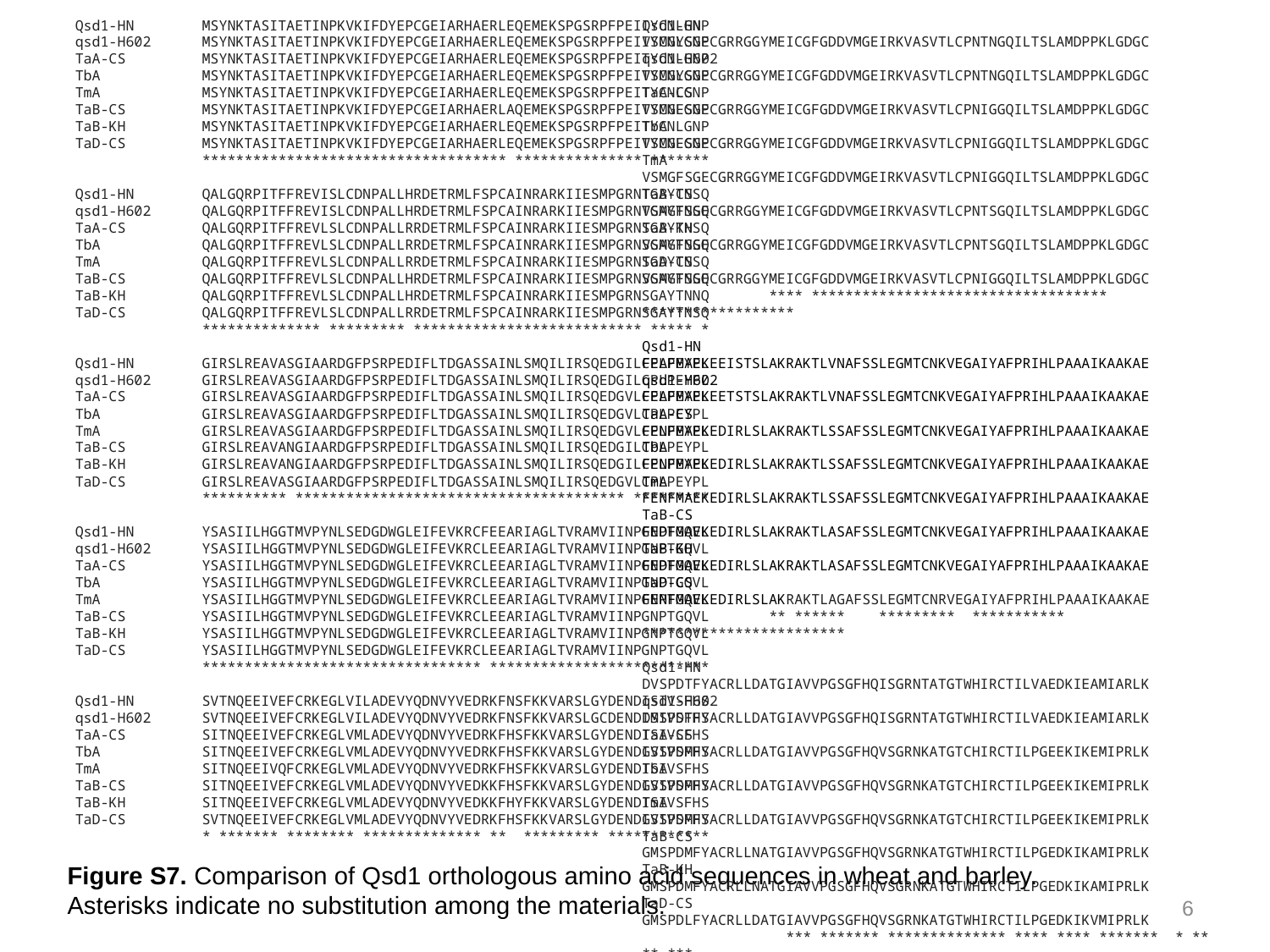

Qsd1-HN MSYNKTASITAETINPKVKIFDYEPCGEIARHAERLEQEMEKSPGSRPFPEIIYCNLGNP
qsd1-H602 MSYNKTASITAETINPKVKIFDYEPCGEIARHAERLEQEMEKSPGSRPFPEIIYCNLGNP
TaA-CS MSYNKTASITAETINPKVKIFDYEPCGEIARHAERLEQEMEKSPGSRPFPEITYCNLGNP
TbA MSYNKTASITAETINPKVKIFDYEPCGEIARHAERLEQEMEKSPGSRPFPEITYCNLGNP
TmA MSYNKTASITAETINPKVKIFDYEPCGEIARHAERLEQEMEKSPGSRPFPEITYCNLGNP
TaB-CS MSYNKTASITAETINPKVKIFDYEPCGEIARHAERLAQEMEKSPGSRPFPEITYCNLGNP
TaB-KH MSYNKTASITAETINPKVKIFDYEPCGEIARHAERLEQEMEKSPGSRPFPEITYCNLGNP
TaD-CS MSYNKTASITAETINPKVKIFDYEPCGEIARHAERLEQEMEKSPGSRPFPEITYCNLGNP
 ************************************ *************** *******
Qsd1-HN QALGQRPITFFREVISLCDNPALLHRDETRMLFSPCAINRARKIIESMPGRNTGAYTNSQ
qsd1-H602 QALGQRPITFFREVISLCDNPALLHRDETRMLFSPCAINRARKIIESMPGRNTGAYTNSQ
TaA-CS QALGQRPITFFREVLSLCDNPALLRRDETRMLFSPCAINRARKIIESMPGRNSGAYTNSQ
TbA QALGQRPITFFREVLSLCDNPALLRRDETRMLFSPCAINRARKIIESMPGRNSGAYTNSQ
TmA QALGQRPITFFREVLSLCDNPALLRRDETRMLFSPCAINRARKIIESMPGRNSGAYTNSQ
TaB-CS QALGQRPITFFREVLSLCDNPALLHRDETRMLFSPCAINRARKIIESMPGRNSGAYTNSQ
TaB-KH QALGQRPITFFREVLSLCDNPALLHRDETRMLFSPCAINRARKIIESMPGRNSGAYTNNQ
TaD-CS QALGQRPITFFREVLSLCDNPALLRRDETRMLFSPCAINRARKIIESMPGRNSGAYTNSQ
 ************** ********* *************************** ***** *
Qsd1-HN GIRSLREAVASGIAARDGFPSRPEDIFLTDGASSAINLSMQILIRSQEDGILCPLPEYPL
qsd1-H602 GIRSLREAVASGIAARDGFPSRPEDIFLTDGASSAINLSMQILIRSQEDGILCPLPEYPL
TaA-CS GIRSLREAVASGIAARDGFPSRPEDIFLTDGASSAINLSMQILIRSQEDGVLCPLPEYPL
TbA GIRSLREAVASGIAARDGFPSRPEDIFLTDGASSAINLSMQILIRSQEDGVLCPLPEYPL
TmA GIRSLREAVASGIAARDGFPSRPEDIFLTDGASSAINLSMQILIRSQEDGVLCPLPEYPL
TaB-CS GIRSLREAVANGIAARDGFPSRPEDIFLTDGASSAINLSMQILIRSQEDGILCPLPEYPL
TaB-KH GIRSLREAVANGIAARDGFPSRPEDIFLTDGASSAINLSMQILIRSQEDGILCPLPEYPL
TaD-CS GIRSLREAVASGIAARDGFPSRPEDIFLTDGASSAINLSMQILIRSQEDGVLCPLPEYPL
 ********** *************************************** *********
Qsd1-HN YSASIILHGGTMVPYNLSEDGDWGLEIFEVKRCFEEARIAGLTVRAMVIINPGNPTGQVL
qsd1-H602 YSASIILHGGTMVPYNLSEDGDWGLEIFEVKRCLEEARIAGLTVRAMVIINPGNPTGQVL
TaA-CS YSASIILHGGTMVPYNLSEDGDWGLEIFEVKRCLEEARIAGLTVRAMVIINPGNPTGQVL
TbA YSASIILHGGTMVPYNLSEDGDWGLEIFEVKRCLEEARIAGLTVRAMVIINPGNPTGQVL
TmA YSASIILHGGTMVPYNLSEDGDWGLEIFEVKRCLEEARIAGLTVRAMVIINPGNPTGQVL
TaB-CS YSASIILHGGTMVPYNLSEDGDWGLEIFEVKRCLEEARIAGLTVRAMVIINPGNPTGQVL
TaB-KH YSASIILHGGTMVPYNLSEDGDWGLEIFEVKRCLEEARIAGLTVRAMVIINPGNPTGQVL
TaD-CS YSASIILHGGTMVPYNLSEDGDWGLEIFEVKRCLEEARIAGLTVRAMVIINPGNPTGQVL
 ********************************* **************************
Qsd1-HN SVTNQEEIVEFCRKEGLVILADEVYQDNVYVEDRKFNSFKKVARSLGYDENDISIVSFHS
qsd1-H602 SVTNQEEIVEFCRKEGLVILADEVYQDNVYVEDRKFNSFKKVARSLGCDENDISIVSFHS
TaA-CS SITNQEEIVEFCRKEGLVMLADEVYQDNVYVEDRKFHSFKKVARSLGYDENDISIVSFHS
TbA SITNQEEIVEFCRKEGLVMLADEVYQDNVYVEDRKFHSFKKVARSLGYDENDISIVSFHS
TmA SITNQEEIVQFCRKEGLVMLADEVYQDNVYVEDRKFHSFKKVARSLGYDENDISIVSFHS
TaB-CS SITNQEEIVEFCRKEGLVMLADEVYQDNVYVEDKKFHSFKKVARSLGYDENDISIVSFHS
TaB-KH SITNQEEIVEFCRKEGLVMLADEVYQDNVYVEDKKFHYFKKVARSLGYDENDISIVSFHS
TaD-CS SVTNQEEIVEFCRKEGLVMLADEVYQDNVYVEDRKFHSFKKVARSLGYDENDISIVSFHS
 * ******* ******** ************** ** ********* ************
Qsd1-HN VSMGYSGECGRRGGYMEICGFGDDVMGEIRKVASVTLCPNTNGQILTSLAMDPPKLGDGC
qsd1-H602 VSMGYSGECGRRGGYMEICGFGDDVMGEIRKVASVTLCPNTNGQILTSLAMDPPKLGDGC
TaA-CS VSMGFSGECGRRGGYMEICGFGDDVMGEIRKVASVTLCPNIGGQILTSLAMDPPKLGDGC
TbA VSMGFSGECGRRGGYMEICGFGDDVMGEIRKVASVTLCPNIGGQILTSLAMDPPKLGDGC
TmA VSMGFSGECGRRGGYMEICGFGDDVMGEIRKVASVTLCPNIGGQILTSLAMDPPKLGDGC
TaB-CS VSMGFSGECGRRGGYMEICGFGDDVMGEIRKVASVTLCPNTSGQILTSLAMDPPKLGDGC
TaB-KH VSMGFSGECGRRGGYMEICGFGDDVMGEIRKVASVTLCPNTSGQILTSLAMDPPKLGDGC
TaD-CS VSMGFSGECGRRGGYMEICGFGDDVMGEIRKVASVTLCPNIGGQILTSLAMDPPKLGDGC
 **** *********************************** ******************
Qsd1-HN FEAFMAEKEEISTSLAKRAKTLVNAFSSLEGMTCNKVEGAIYAFPRIHLPAAAIKAAKAE
qsd1-H602 FEAFMAEKEETSTSLAKRAKTLVNAFSSLEGMTCNKVEGAIYAFPRIHLPAAAIKAAKAE
TaA-CS FENFMAEKEDIRLSLAKRAKTLSSAFSSLEGMTCNKVEGAIYAFPRIHLPAAAIKAAKAE
TbA FENFMAEKEDIRLSLAKRAKTLSSAFSSLEGMTCNKVEGAIYAFPRIHLPAAAIKAAKAE
TmA FENFMAEKEDIRLSLAKRAKTLSSAFSSLEGMTCNKVEGAIYAFPRIHLPAAAIKAAKAE
TaB-CS FEDFMAEKEDIRLSLAKRAKTLASAFSSLEGMTCNKVEGAIYAFPRIHLPAAAIKAAKAE
TaB-KH FEDFMAEKEDIRLSLAKRAKTLASAFSSLEGMTCNKVEGAIYAFPRIHLPAAAIKAAKAE
TaD-CS FENFMAEKEDIRLSLAKRAKTLAGAFSSLEGMTCNRVEGAIYAFPRIHLPAAAIKAAKAE
 ** ****** ********* *********** ************************
Qsd1-HN DVSPDTFYACRLLDATGIAVVPGSGFHQISGRNTATGTWHIRCTILVAEDKIEAMIARLK
qsd1-H602 DMSPDTFYACRLLDATGIAVVPGSGFHQISGRNTATGTWHIRCTILVAEDKIEAMIARLK
TaA-CS GVSPDMFYACRLLDATGIAVVPGSGFHQVSGRNKATGTCHIRCTILPGEEKIKEMIPRLK
TbA GVSPDMFYACRLLDATGIAVVPGSGFHQVSGRNKATGTCHIRCTILPGEEKIKEMIPRLK
TmA GVSPDMFYACRLLDATGIAVVPGSGFHQVSGRNKATGTCHIRCTILPGEEKIKEMIPRLK
TaB-CS GMSPDMFYACRLLNATGIAVVPGSGFHQVSGRNKATGTWHIRCTILPGEDKIKAMIPRLK
TaB-KH GMSPDMFYACRLLNATGIAVVPGSGFHQVSGRNKATGTWHIRCTILPGEDKIKAMIPRLK
TaD-CS GMSPDLFYACRLLDATGIAVVPGSGFHQVSGRNKATGTWHIRCTILPGEDKIKVMIPRLK
 *** ******* ************** **** **** ******* * ** ** ***
Qsd1-HN AFHESFMNEFRDRS
qsd1-H602 AFHESFMNEFRDRS
TaA-CS EFHESFMNEFRDRS
TbA EFHESFMNEFRDRS
TmA EFHESFMNEFRDRS
TaB-CS EFHESFMNEFRNRS
TaB-KH EFHESFMNEFRNRS
TaD-CS EFHESFMNEFRDRS
 ********** **
Figure S7. Comparison of Qsd1 orthologous amino acid sequences in wheat and barley. Asterisks indicate no substitution among the materials.
6

## Slide 7
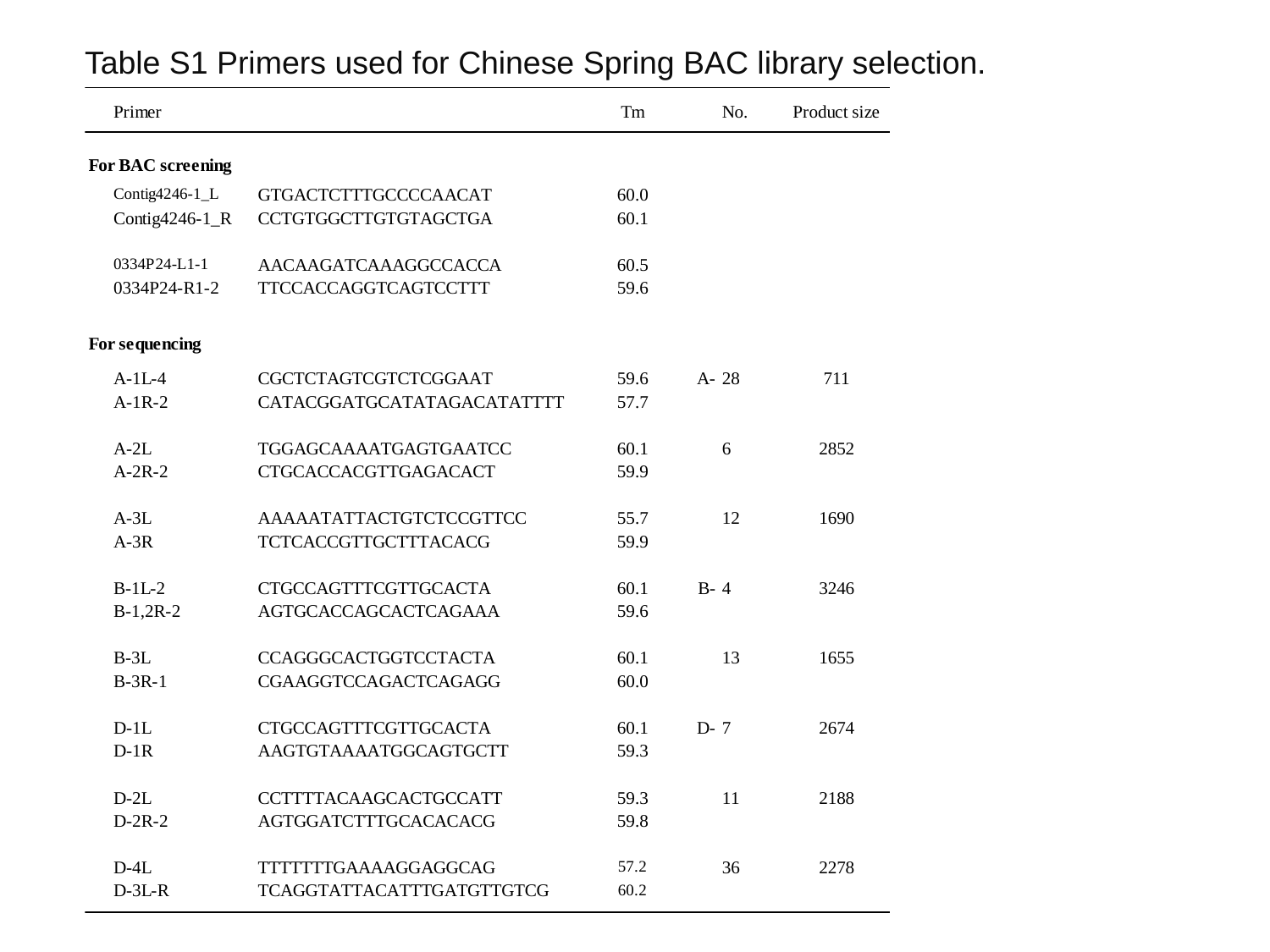

Table S1 Primers used for Chinese Spring BAC library selection.

## Slide 8
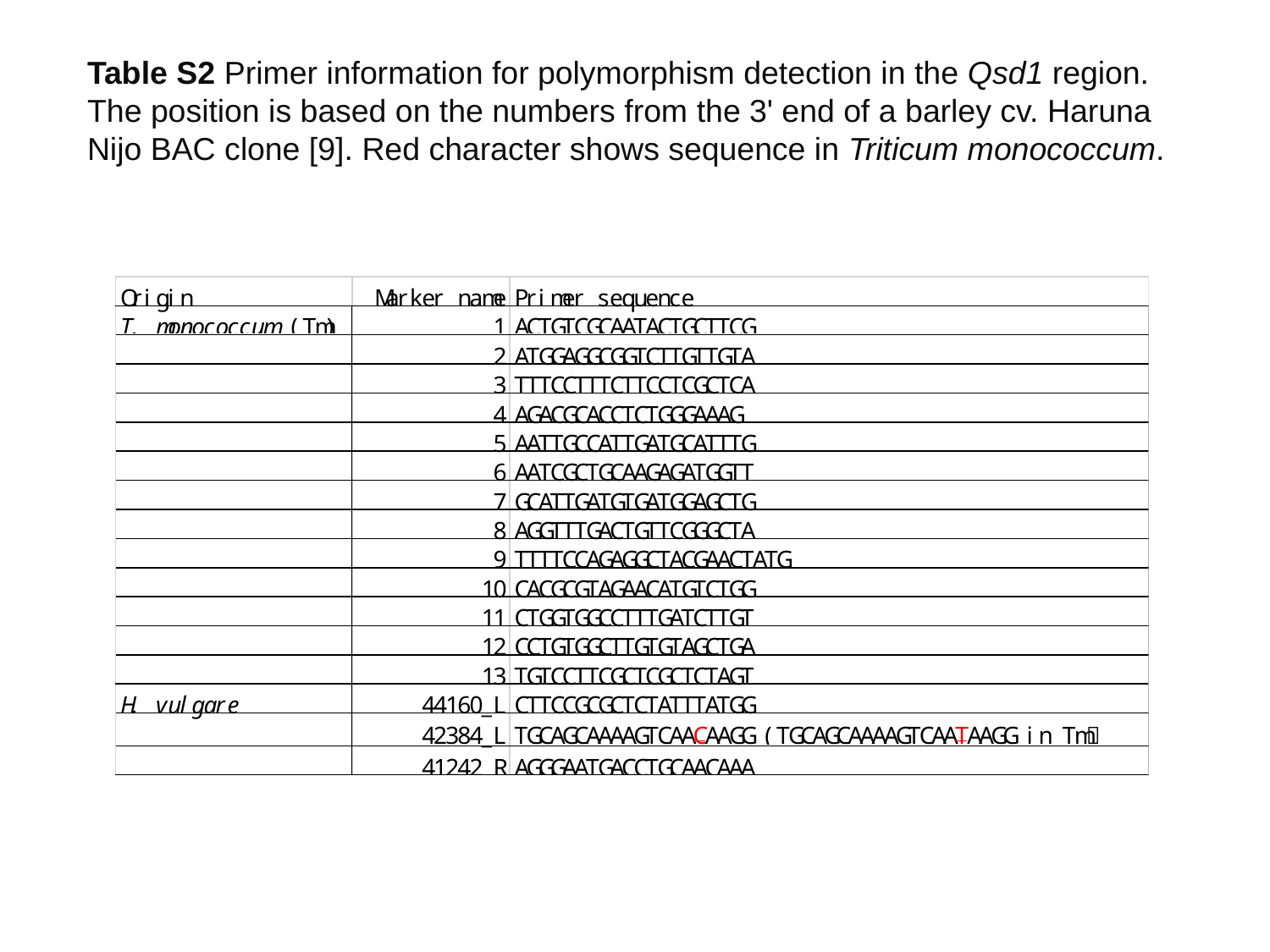

Table S2 Primer information for polymorphism detection in the Qsd1 region. The position is based on the numbers from the 3' end of a barley cv. Haruna Nijo BAC clone [9]. Red character shows sequence in Triticum monococcum.
