# Supplementary material for: Sequence differences in the seed dormancy gene Qsd1 among various wheat genomes
Source: BMC Genomics. 2017 Jun 29;18:497. doi: 10.1186/s12864-017-3880-6 (PMC5492916; doi:10.1186/s12864-017-3880-6)
Supplement: Supplementary file 2 — Nucleotide sequences of Qsd1 orthologous loci in the sub-genomes of cv. Chinese Spring (TaA-CS, TaB-CS, and TaD-CS). The cDNA sequences are underlined. Start and stop codons are shown in bold. Introns are shown in standard font. b Nucleotide sequences of Qsd1 orthologs in the A sub-genomes of cv. Chinese Spring (TaA-CS), Triticum boeoticum (TbA), and T. monococcum (TmA). The cDNA sequences are underlined. Polymorphisms between the A sub-genome vs. diploid wheat and within diploid wheat accessions are highlighted in green and purple, respectively. Start and stop codons are shown in bold. The primer positions used for sequencing are highlighted in gray and marker names are shown in parentheses. (ZIP 43 kb) [file 12864_2017_3880_MOESM2_ESM.zip › Supplementary Material 2 (Figure S3a).docx]

**Figure S3a.** Nucleotide sequences of *Qsd1* orthologous loci in the sub-genomes of cv. Chinese Spring (TaA-CS, TaB-CS, and TaD-CS). The cDNA sequences are underlined. Start and stop codons are shown in bold. Introns are shown in standard font.

TaB-CS ACCCAGCACGGCACAATCACTCACTCTCTGCATCCTCCCCTCCCCTCTCCTCTGCGCCAC

TaA-CS -------------CAATCACTCACTCTCTGC-----CTCCTCCCATTTCCTCTGCGCCAC

TaD-CS -------ACAGCACAATCACTCACTCTCTGCATCCTCCCCTCCCCTCTCCTCTGCGCCAC

****************** * ****** * *************

TaB-CS CTTAGCCAAGCTCTCCTCCTTCCTTCCCCGCCCGCGCGCGCTCTCTCCTCGCCGGCGCCG

TaA-CS CTTAGCCAAGCTCTCCTCCTTCCTTC----CCCGCGCGCGCTCTCTCCTCGCCGGCGCCG

TaD-CS CTTAGCCAAGCTCTCCTCCTTCCTTC----CCCGCCTGCGTTCTCTCCTCGCCGGCGCCG

************************** ***** *** *******************

TaB-CS CCCCGCGCC**ATG**TCGTACAACAAGACCGCCTCCATCACCGCCGAGACCATAAACCCCAAG

TaA-CS CCCCGCGCC**ATG**TCGTACAACAAGACCGCCTCCATCACCGCCGAGACCATAAACCCCAAG

TaD-CS CCCCGCGCC**ATG**TCGTACAACAAGACGGCGTCCATCACCGCGGAGACCATAAACCCCAAG

************************** ** *********** ******************

TaB-CS -----GTACCGTACCGTACCTGACGGACGCCTCCCCCCCTGCTCCTGGTGCTTG-CCCTT

TaA-CS ---------------GTACCTGACGGACGCCTCCTCA------TCCGGTGCTTG-CCCTG

TaD-CS GTACCGTACCGTACCGTACCTGACGGACGCCTCCTCA------TCCGGTGCTTGCCCCTT

******************* * * ******** ****

TaB-CS CTTCACGTACGCCAAACCTGACTCGGTTCTCCATGTTTTTCTTTCTTCCTCACTCGCAGG

TaA-CS ATTCACGTACGCCAAACCTGACTTGATTCTCCATGTTTTCCTTTCTTCCTCGCTCACAGG

TaD-CS CTTCACCTACGCCAAACCTGA-----TTCTCCATGTTTTTCTTCCTTCCTCGCTCACAGG

***** ************** ************* *** ******* *** ****

TaB-CS TGAAGATCTTCGACTACGAGCCCTGCGGGGAGATCGCCAGGCACGCAGAGGTAACAGCAA

TaA-CS TGAAGATCTTCGACTACGAGCCCTGCGGGGAGATCGCCAGGCACGCAGAGGTAACAGCAA

TaD-CS TGAAGATCTTCGACTACGAGCCCTGCGGAGAGATCGCCAGGCACGCAGAGGTAACAGCAA

**************************** *******************************

TaB-CS CAGTGCTGCCTGCGCTCACCCCCCGCCGTTCATCTGTCCATGTATGTATGTATGTATGTA

TaA-CS CAATTCTACCTGCACTCA-CCCTCGCCGTTCAACTGTCCATGTATGTA------------

TaD-CS CAATGCTACCTGCACTCA-CCCCCGCCGTTCAACTGTCCATGTATGTA------TCAGTA

** * ** ***** **** *** ********* ***************

TaB-CS TGTATGTGTCGC---AGTCCAGTCAGACTGTCTGTCTATCGACACAGCCAGTGGCAGTGG

TaA-CS TGTATGTGTCGCAGTAGTCCAGTCAGACTGTCTGTCTATGGACACAGTCAGTGG------

TaD-CS TGTATGTGTCGCAGTAGTCCAATCAGACTGTCAATCG------ACAGTCAGTGG------

************ ****** ********** ** **** ******

TaB-CS CCAGTGGGTTACCTCTGACATAGTAGTACCCGAGTGCACAAGCTAACTAAAGAAATGACT

TaA-CS -CAGTGGGTTACCCCTGACCTAGTAGTACCCGGGTGCGCAAGCTAACTAAAGAAATGACT

TaD-CS -CAGTGGCTTACCCCTGACCTAGTAGTACCCGAGTGCACAACCTAACTAAAGAAATGAGT

****** ***** ***** ************ **** *** **************** *

TaB-CS GCTGTAAACTGAAACTAAAGTAGCGCACAAGCCAACTAAAGAAATGACTTCTGTAAACTG

TaA-CS TCTGTAAACTGAAACC-AAGTAGTGCACAAGCCAACTAAAGAAATGACTTCTGTAAACTG

TaD-CS TCTGTAAACTGAAACT-AAGTAGTGCACAAGCCAACTAAAGAAATGACTTCTGTAAACTG

************** ****** ************************************

TaB-CS AAACTAAGTGCTAGACTGCATTTTGCACTACAGCCAGTTCAGATGATGCATCCAACTTTG

TaA-CS AAACTAAGAGCTAGACTGCATTTTGCACTACAGCCAGTTCAGATGATGCATCCAACTTTG

TaD-CS AAACCAAGTGCTAGGCTGCATTTTGCACTACAGCCAGTTCAGATGATGCATCCAACTTTG

**** *** ***** *********************************************

TaB-CS ATCTTTGGTATCATCTGGCATTCCTGGCCCATAATAAATCTGCAAAAACACTGGTTTGAC

TaA-CS ATCTTTGGTATCATCTGACATTCCTGGCCC---ATAAATCTGCAAAAGCACTGGTTTGAC

TaD-CS ATCTTTGGTATCATCGGACATTCCTTGCCC---GTAAATCTGAAAAAGCACTGGTTTGAC

*************** * ******* **** ******** **** ************

TaB-CS CGTACAAGTTTCCTATCGGCATATGCCGGTTTTCGTGTTATCCAGCGGTTGGCGCAGGAG

TaA-CS TGTACAAATTTTCTTTCGGCATATGCCGGTTTTCGTGTTATCCAGCGGTTGGAGCAGGAG

TaD-CS TGTACAATTTTCCTTTCGGCATATGCCGGTTTTCGTGTTATCCAGAGGTTGGAGCAGGAG

****** *** ** ****************************** ****** *******

TaB-CS ATGGAGAAGAGCCCAGGTTCTCGCCCTTTTCCAGAGGTGCGTCTTTGTTTACATATATAC

TaA-CS ATGGAGAAGAGCCCCGGTTCTCGCCCTTTCCCAGAGGTGCGTCTTTGTTTACATACA---

TaD-CS ATGGAGAAGAGCCCAGGTTCTCGCCCTTTTCCAGAGGTGCGTCTTTGTTTACATACATAT

************** ************** ************************* *

TaB-CS TTATGTTCAATCCTGGCCAAAATCACTAATCGATGATGATGAGTTGCTTCCTTGCAGATA

TaA-CS ATGTATTCAATCCTGGCCAAAATCACTAGTCAATGATGATAAGTTGCTTCCTTGCAGATA

TaD-CS ATATGTTCAATCCTGGCCAAAAACACTAATCGATGATGATGAGTTGCTTCCTTGCAGATA

* * ***************** ***** ** ******** *******************

TaB-CS ACATACTGCAACCTTGGGAACCCCCAGGCTCTCGGCCAGCGACCCATAACCTTCTTCCGT

TaA-CS ACATACTGCAACCTTGGGAACCCCCAGGCTCTCGGCCAGCGACCCATAACCTTCTTCCGT

TaD-CS ACATACTGCAACCTTGGGAACCCCCAGGCTCTCGGCCAGCGACCCATAACCTTCTTCCGT

************************************************************

TaB-CS GAGGTTATAACCCTTAATCACACTCTCCAAAAAAAAAACTTAATCTGGGAGGC----ATT

TaA-CS GAGGTTACAACCTTTAATCACACTCTCCAAAAAATA---TTAATCCGGGAGGCATTAATT

TaD-CS GAGGTTACAACCCATAATCACACTCTTCAAAAAAA-----TAATCTGGGAGGCATTAATT

******* **** ************ ******* ***** ******* ***

TaB-CS GCTGTCTTATGCCTCAAATATCATCATCTGCCTTCAGGTTCTTTCTCTGTGTGACAATCC

TaA-CS GCTGTCTTATGCCTCAAATATCATCATCTGCCTTCAGGTTCTTTCCCTGTGCGACAATCC

TaD-CS GCTACCTTATGCCTCAAATATCATCATCTGCCTTCAGGTTCTTTCCCTGTGCGACAATCC

*** **************************************** ***** ********

TaB-CS AGCTCTCCTGCACAGGGATGAAACTCGTATGCTATTCAGGTTTGTTGGGCTTAAATAATA

TaA-CS AGCTCTCCTGCGCAGGGATGAAACTCGTATGCTATTCAGGTTTGTTGGGCTTAAATAATA

TaD-CS AGCTCTCCTGCGCAGGGATGAAACTCGTATGTTATTCAGGTTTGTTGGGCTTGAATAATA

*********** ******************* ******************** *******

TaB-CS CTGTACTACTAGCTCAGAGTCTGTTTACCCACCTCTCACAATTTTCTGTTCAAAAGAAAA

TaA-CS CTGTACTGCTAGCTCAAAGTCTGTTTTCCCATCTCTCACAATTTTCTCTTCAGAAGAAAA

TaD-CS CTGTACTACTAGCTCAAAGTCTGTTTACCCATCTCTCACAATTTTCTGTTCAAAAGAAAA

******* ******** ********* **** *************** **** *******

TaB-CS GCAAATTCTTCAGCCATTGATGAGTTTGCTGTCAGTTCTAGCACAAAAGTTGC---TAGT

TaA-CS GCAAATT-----GCCATTGATGCATTTGCTTTCAGTTCTAGCAGAAAAGTTGCTAGTAGT

TaD-CS GCAAAGTCTTCAGCCATTGATGCGTTTGCTTTCAGTTCTAGCAGAAAAGTTGGTAGTAGT

***** * ********** ****** ************ ******** ****

TaB-CS ATCATGCACCATCTTGCTGTGACGATTTGATTCGTCGAAACCTGACCAGTGTTGTTTCAA

TaA-CS ATCATGCACCATCTTGCTGTGACGATTTGATTCGTCGAAACCTGACCAGTATTGTTTCAA

TaD-CS ATCAAGTACCATCTCTCTGTGACGATTTAATTCGTCGAAACCTGACAGATATTGTTTCAA

**** * ******* ************ ***************** * *********

TaB-CS CCTGCTATTACAGCCCATGTGCCATAAATAGAGCGCGGAAGATTATTGAGTCCATGCCCG

TaA-CS CCTGCTATTACAGCCCATGTGCCATAAATAGAGCGCGGAAGATTATTGAGTCCATGCCTG

TaD-CS CCTGCTATTACAGCCCATGTGCCATAAATAGAGCGCGGAAGATTATTGAGTCCATGCCTG

********************************************************** *

TaB-CS GCAGAAACTCTGGTGCATATACTAACAGTCAGGTATTACATTGGAATACCAAGATAATCT

TaA-CS GCAGAAACTCCGGTGCATATACTAACAGTCAGGTATTACATTGGAATACCAAGATAATCT

TaD-CS GCAGAAACTCTGGTGCATATACTAACAGTCAGGTATTACAT-------------------

********** ******************************

TaB-CS TACAATCAGCTGCATAAACTAGAGTCGTCTGATGTTTCGACTTTTGAGGCGATGCCGCAT

TaA-CS TACAACCAGGTGC---------------CTGATGTTTCGACTTTTGGGGCGGTGCCGCAT

TaD-CS ------------------------------------------------------------

TaB-CS TAGTGCACCAGCACTCAGAAAGAGATCACCACTTAGTAGGACCAGTGCCCTGGCCTTGGT

TaA-CS TAGTGCACCAACA----------------------------------CCCTGGTCTTGGT

TaD-CS -------------------------------------------------------TTGAT

*** *

TaB-CS GTTGTCGTCTTGTCCGACGATG-ATAATATTTTTCCCATAC-------------------

TaA-CS GTTGTTGTCTTGTCCGGCGATG-ATAATATTTTTCCCATACTATTCTGCACCACGTTGAG

TaD-CS GTTGTCGTCTTGTCCGACGATGAATAATATTTTTCCCATGC-------------------

***** ********** ***** **************** *

TaB-CS ----------------------------------------------------------TA

TaA-CS ACACTTATTTTGGGACGGACGGAGTATTTAGGAACGGAGACAGTAATATTTTTTTCCATA

TaD-CS ------------------------------------------------------------

TaB-CS TTATTCTGCACCTCGTTGTTGTAAACTCCTCTAACATGATCAGGGAATCAGAAGTTTGCG

TaA-CS TTATTCTGCACCACGTTGTTGTAAACTCCTCTAACATGATCAGGGAATCAGAAGTTTGCG

TaD-CS -TATTCTGCACCACGTTGTTGTGAACTCCTGTAACATGATCAGGGAATCAGAAGTTTGCG

*********** ********* ******* *****************************

TaB-CS GGAAGCAGTTGCAAATGGAATCGCTGCAAGAGATGGTTTTCCATCAAGACCAGAGGACAT

TaA-CS CGAAGCAGTCGCAAGTGGAATCGCTGCAAGAGATGGTTTTCCATCAAGACCAGAAGACAT

TaD-CS CGAAGCAGTCGCAAGTGGAATCGCTGCAAGAGATGGTTTTCCATCAAGACCAGAAGACAT

******** **** *************************************** *****

TaB-CS CTTTCTGACAGATGGAGCGAGTTCAGCCGTAATACTTTACATTCAACAGCTCGATCACAT

TaA-CS CTTTCTGACAGATGGAGCGAGTTCAGCCGTAATACTCTACATTCAACAGCTCCATCACAT

TaD-CS CTTTCTGACAGATGGAGCGAGTTCAGCCGTAATACTCTACATTCAACAGCTCCATCACAT

************************************ *************** *******

TaB-CS CAATGCATTTCGTCTATCTCGATATGCTAAATTGGGCTGCATTTTTCATTTCTTCCTTCC

TaA-CS CAATGCACTTCGTCTATCTCGACGTGCTAAATTGGGCTGCATTTTTCATTTCTTCCTTCC

TaD-CS CAATGCACTTCGTCTATCTTGACATGCTAAATTGGGCTGCATTTTTCATTTCTTCCTTCC

******* *********** ** ************************************

TaB-CS AGATTAATTTGAGTATGCAGATACTCATTAGGTCCCAAGAAGATGGCATTCTATGCCCTT

TaA-CS AGATTAATTTGAGTATGCAGATACTCATTAGGTCCCAAGAAGATGGCGTTTTATGCCCTT

TaD-CS AGATTAATTTGAGTATGCAGATACTCATTAGGTCCCAAGAAGATGGTGTTTTATGCCCTT

********************************************** ** *********

TaB-CS TACCTGAATATCCGTTATACTCGGCGTCCATTATACTTCATGGTGGGACTATGGTATGGT

TaA-CS TACCTGAATATCCGTTATACTCGGCGTCCATTATACTTCATGGTGGGACTATGGTATGGT

TaD-CS TACCTGAATATCCGTTATACTCGGCGTCCATTATACTTCATGGTGGGACTATGGTATGGT

************************************************************

TaB-CS GCTAGCTTTATGAAATAATTGATCAGCGGTCGCAATTCTTCAGTACTT-TTTTTTTCATT

TaA-CS GCTAGCTTTATGAAATAATTGATCGGCGGTCGCAATTCTTCAGTACTTAATTTGTTCATT

TaD-CS GCTAGCTTTATGAAATAATTGATCGGCGGCCGCAATTCTTCAGTACTTAATTTGTTCATT

************************ **** ****************** *** ******

TaB-CS GCATCTCAAACCTATAGATCAGATTCCTGACATTTTTTACTTAAAAAGTGT---------

TaA-CS GCATCTCAAACCTATAGATCAGATTCCTGACA-TTTTTACATAAAAAGTGT---------

TaD-CS GCATCTCAAACCTATAGATCAGATTCCTGACA-TTTTTTCTTATAAAGTGTAAAATGGCA

******************************** ***** * ** *******

TaB-CS ---------AAAGGACTGCTCATTTTATTACATGCAAGAAACGACCAAGTTACTAAATTT

TaA-CS ---------AAAGGACTGCTCATTTTATTACATGCAAGAAATGACCAAGTTACTGAATTT

TaD-CS GTGCTTGTAAAAGGTCTGCTCATTTTATTACATGCAAGAAATGACCAAGTTACTGACTTT

***** ************************** ************ * ***

TaB-CS GGTTTGTAGGTACCATACAATCTTAGTGAGGACGGTGATTGGGGGCTTGAGATCTTCGAA

TaA-CS GGTTTGTAGGTGCCATACAATCTTAGTGAGGACGGTGATTGGGGGCTTGAGATCTTCGAA

TaD-CS GGTTTGTAGGTACCATACAATCTTAGTGAGGACGGTGATTGGGGGCTTGAGATCTTCGAA

*********** ************************************************

TaB-CS GTAAAGAGGTGCTTGGAGGAGGCACGCATCGCAGGTTTGACTGTTCGGGCTATGGTGATC

TaA-CS GTAAAGAGGTGCTTGGAGGAGGCACGCATCGCAGGTTTGACTGTTCGGGCTATGGTGATC

TaD-CS GTAAAGAGGTGCTTGGAGGAGGCACGCATCGCAGGTTTGACTGTTCGGGCTATGGTGATC

************************************************************

TaB-CS ATAAACCCCGGAAATCCGACGGGACAGGCACGCAAACAGATATTTATGTACCTTGCAATA

TaA-CS ATAAACCCCGGAAATCCGACGGGACAGGTACGCAAACAGATATTTATGTACCTTGCAATA

TaD-CS ATAAACCCCGGAAATCCGACGGGACAGGTATGCAAACAGATATTTATGTACCTTGCAATA

**************************** * *****************************

TaB-CS CCATAAATGTGA----ATGGGATAAAACAAAACA-AAAAATCTAGAAGGTTCCCTAAATT

TaA-CS CCATAAATGTGAATGCATGGGATAAAACAAAA---AAATATCTAGAAGGTTCCCTAAAAT

TaD-CS CCATAAATGTGA----ATGGGATAAAACAAAAAATATATATCTAGAAGGTTCCCTAAAAT

************ **************** * * ******************* *

TaB-CS AATGCTGAAACTCAACATCTGCAAGCTCTGCTGTAGGTACTGTCTATCACCAACCAGGAG

TaA-CS ACTGCTGAAACTCAACATCTGCAAGCTCTGCTGTAGGTACTGTCTATCACCAACCAGGAG

TaD-CS ACTGCTGAAACTCAACATCTGCAAGCTCTGCTGTAGGTACTGTCTGTCACCAACCAGGAG

* ******************************************* **************

TaB-CS GAGATAGTAGAATTTTGTCGGAAAGAAGGTTTGGTTATGCTTGCCGATGAGGTTTGCACT

TaA-CS GAGATAGTAGAATTTTGTCGGAAAGAAGGTTTGGTTATGCTTGCCGATGAGGTTTGCACT

TaD-CS GAGATAGTAGAATTTTGTCGGAAAGAAGGTTTGGTTATGCTTGCTGATGAGGTTTGCACT

******************************************** ***************

TaB-CS TGGTGAGCTGCAGTAGTTTTGTGACGCGGACGCGTGTGTAGACATCCTACATTTGTGTAA

TaA-CS TGGTGAGCTGCAGTAGTTTCCTGACGCGGACGCGTGTGTAGACATCCTACATTTGTGTAA

TaD-CS TGGTGAACTGCAGTAGTTTTGTGACGCGGACGCGTGTGTAGACATCCTACATTTGTATAA

****** ************ *********************************** ***

TaB-CS ATGTATTTACAGGTATACCAAGATAACGTCTATGTGGAGGATAAGAAATTCCATTCTTTC

TaA-CS ATGTATTTACAGGTATACCAAGATAACGTCTATGTGGAGGATAGGAAATTTCATTCTTTC

TaD-CS ATGTATTTACAGGTATACCAAGATAACGTCTATGTGGAAGATAGGAAATTCCATTCTTTC

************************************** **** ****** *********

TaB-CS AAGAAAGTAGCCAGATCACTTGGGTATGACGAGAATGACATCTCCATAGTGTCATTTCAC

TaA-CS AAGAAAGTAGCCAGATCACTTGGGTATGACGAGAATGACATCTCCATAGTGTCATTTCAC

TaD-CS AAGAAAGTAGCCAGATCACTTGGGTATGACGAGAATGACATCTCCATAGTGTCATTTCAC

************************************************************

TaB-CS TCGGTCTCGATGGGTAAACCGCTGTTCATCAGAAACATAGTTCATAGCCTCTGGAAAATA

TaA-CS TCAGTCTCAATGGGTAAACCGTTGTTCATCAGAAACATAGTTCGTAGCCTCTGGAAAATA

TaD-CS TCGGTCTCGATGGGTAAACCGTTGTTCATCAGAAACATAGTTCATAGCCTCTGGATAATA

** ***** ************ ********************* *********** ****

TaB-CS AGCCTTAGAGCAG------ATGGTTATTTTTTCATCATCTGAACTTGACAGGGTTCTCTG

TaA-CS AGCCTTAGAGCAGAGACTAATGGTTATTTTTTCATCTTCTGAACTTGACAGGGTTCTCTG

TaD-CS AGCCTTAGAGCAGGGACTAATGGTTATTTTTTCATCATCTGAACTTAACAGGGTTCTCTG

************* ***************** ********* *************

TaB-CS GAGAATGTGGCAGAAGGGGAGGCTACATGGAGATATGTGGTTTTGGAGATGATGTGATGG

TaA-CS GAGAATGTGGCAGAAGGGGAGGCTACATGGAGATATGTGGTTTTGGAGATGATGTGATGG

TaD-CS GAGAATGTGGCAGAAGGGGAGGCTACATGGAGATATGTGGTTTTGGAGATGATGTTATGG

******************************************************* ****

TaB-CS GTGAGATTCGCAAAGTGGCTTCTGTGACTCTTTGCCCCAACACAAGTGGTCAAATTCTTA

TaA-CS GTGAGATTCGCAAAGTGGCTTCCGTGACTCTTTGCCCCAACATAGGTGGTCAAATTCTTA

TaD-CS GTGAGATTCGCAAAGTGGCTTCCGTGACTCTTTGCCCCAACATAGGTGGTCAAATTCTTA

********************** ******************* * ***************

TaB-CS CTAGCCTTGCTATGGATCCACCGAAGGTTCGTTCGTCCTGCCTTTCTTGTGTTTGTGATA

TaA-CS CTAGCCTTGCTATGGATCCACCGAAGGTTTGTTCGTCCTGCCCTTCTTGTGTTTGTTATA

TaD-CS CTAGCCTTGCTATGGATCCACCGAAGGTTCGTTCGTCCTGCCTTTCTTGTGTTTGTTATA

***************************** ************ ************* ***

TaB-CS ATTTATTTTAATTACTACAACCTCCGTCCCAAATTACTTGTCTTAGATTTATCTAGATAC

TaA-CS ATTTATTTTAATTACTACAACCTCCGTCCCAAATTACTCGTCTTAGATTTGTCTAGATAC

TaD-CS ATGTATTTTAATTACTACAACCTCCGTCCCAAATTACTTGTCTTAGATTTGTCTAGATAC

** *********************************** *********** *********

TaB-CS GGATGTATCTAGACACTAAAACATGTTAGATACATCCATATCTAGAGAAATCTAAGACGA

TaA-CS TGATGTATCTA-ACAGTAAAACGTGTC---TGTATCTTTATCTAGACAAATCTAAGACAA

TaD-CS GGATGTATCTA---------------------------------GACAAATCTAAGACAC

********** ** ***********

TaB-CS GTAATTCGGGGTGGAGGGAGTATATCTTATCTTATCCTGAAAGCATTGTAAAGCTGCCAT

TaA-CS GTAATTCAGGACGGAGGGAGTATATCTTGTCTTATCCTGAAAGCATTGTAAAGCTGCCAT

TaD-CS GTAATTCAGGACGGAGGGAGTATATCTTATCTTATCCTGAAAGCATTGTAAAGCTGCCAT

******* ** **************** *******************************

TaB-CS CCTTTATCGGTAATGCTAGCTGGTCAAACGTTCGCTGGGAAGGAACTTCCAGTGAATGCC

TaA-CS CCTTCATCGGTAATGCTAGCTGGT-GAACCTTCGCTGGGAAGGAACTCCCAGTGAACACC

TaD-CS CCTTTATCGGTAATGCTAGCTGGT-GAACATTCGCTGGGAAGGAACTCCCAGTGAATGCC

**** ******************* *** ***************** ******** **

TaB-CS CTCGCAGTCGTTTGATCGAGATCAAACAATGACAG-TTTTTTGAAAAAAATGCCTTTTCT

TaA-CS CTCGCAGTCGTTTGATCAAGATCAAACG-TGACAGTTTTTTTGAAAATTTTGCCTTTTCT

TaD-CS CTGGCAGTCGTTTGATCAAGACCAAACGATGACAG-TTTTTTGAAAGAATTGTCTTTTCT

** ************** *** ***** ****** ********** ** *******

TaB-CS AGGAAGGAATTGTCATACTAGTCCGAAGAAATTGCTG-----CCCCCATATAACTAAAAT

TaA-CS AGGAAGGAATTGTCATACTAGTCTGAAGAAATTGCTG-----CCTCCCTATAACTAAAAT

TaD-CS AGGAAGGAATTGTCATACTAGTCTGAAGAAATTGCTGCCCCCCCCCCCTATAACTAAAAT

*********************** ************* ** ** ************

TaB-CS TGCCATGGAAATCGTTCGC-AATGTCGTCCTCCCCAGCGAACCTTCGCCAGCTATTGAAG

TaA-CS TG-CATGGAAATCATACGCAAATGCCATCCCCCCCAACGAACCTTCGCTAGCTATTGAAG

TaD-CS TGCCATGGAAATCGTTCACAAATGCCATCCT-CCCAGCGAACCTTCGCTAGCTGTTGAAG

** ********** * * * **** * *** **** *********** **** ******

TaB-CS TCCTTACATGATAGTACCCAA-----------------ATAATTATTTTCAGCAAAAAAA

TaA-CS TCCTTACATGACAGTACCCAAAAAATGTCAAAAAGATTATAATTATTTTCAGCAAAAAAA

TaD-CS TCCTTACATGACAGTACTCAAAAGAATTCAAAAAGATTATAATTATTTTCAGCAAAAAAA

*********** ***** *** **********************

TaB-CS A------------------------TCCTTATTGACTTTTGCTGCAAGTTTCTTGGGCCG

TaA-CS ATCCTTATTGACTTTTTTAAATAAGTCCTTATTGACTTTTGCTGCAAGTTTCTTGGGCTG

TaD-CS T------------------------TCCTTATTGACTTTTGCTGCAAGTTTCTTGGGCCG

********************************* *

TaB-CS TTTTTATTAGGGTATTATTGATGTAATTGTTCCCACCATTGGCATACTTGTAATCAAAAT

TaA-CS TTTTTATTGGGTTATTATTAATGTAATTATTCCTACCATTCGCATACTTGTAATCAAAAT

TaD-CS TTTTTATTAGGTTATTATTAATGTCATTATTCCCACCATTCGCATACTTGTAATCAAAAT

******** ** ******* **** *** **** ****** *******************

TaB-CS TGATTTGATGCAGCTTTGATGAGATCTTCGTTCTGCTTTTTAGATAAGAGGTTTTTGTGT

TaA-CS TGATTTGATGCAGCTTTGATGAGATTTTCGTTCTGGTTTTTAGACAAGAGGTTTTTGTGT

TaD-CS TGATTTGATGCAGCTTTGATGAGATTTTCGTTCTGGTTTTTAGATGAGAGGTTTTTGTGT

************************* ********* ******** **************

TaB-CS CCTTTGCAATATTTGTTGCCCCTTAACACTTACTGCCTCAATTTTCTCACAGAATAGACA

TaA-CS CCTTTGCAATATTAGTTGCCCGTTGACCCTTGCTGCCTCAATTTTGTCACAAAATAGACA

TaD-CS CCTTTGCAATATTAGTTGCCCCTTAACCCGTGCTGCCTCAATTTTCTCACAAAATAGACA

************* ******* ** ** * * ************* ***** ********

TaB-CS ACCCCCCACCTTGAAAAGAATAGATATGCATGTGTTTGTTTTTACCAAACTCATCTTGAT

TaA-CS ACCCCCTACCTAGAAAAGAATAGATATGTATGTGTTTGTTTCTACCAAACTCATCTTGAT

TaD-CS ACCCTCCACCTAGAAAAGAATAGATATGCACGTGTTTGTTTTTACCAAACTCATCTTGAT

**** * **** **************** * ********** ******************

TaB-CS ATTTGCAGCTGGGAGATGGTTGTTTTGAGGATTTTATGGCTGAAAAGGAAGACATCCGTT

TaA-CS ATTTGCAGCTGGGAGATGGTTGTTTTGAGAATTTTATGGCTGAAAAGGAAGACATCCGTT

TaD-CS ATTTGCAGCTGGGAGATGGTTGTTTTGAGAATTTTATGGCTGAAAAGGAAGACATCCGTT

***************************** ******************************

TaB-CS TATCTCTCGCCAAGCGCGCCAAGGTA---TCCTCCTACGTACATGTTGTCAACCAGCACG

TaA-CS TATCTCTCGCCAAGCGCGCCAAGGTATCCTCCTCCTACATACACATTGTCAACAAGCACA

TaD-CS TATCTCTCGCCAAGCGCGCCAAGGTATCCTCCTCCTACATACGCACTG------------

************************** ********* *** **

TaB-CS CCATCTTTCTTCTTCCTCCTCTATGAGAGGAATGATGAAATGACTCAGTGAGA--CAAGT

TaA-CS CCATCTTTCTTCTT---CCTCTATCAGAGGAATGATGAAATGACTCAGTGAGACACAAGT

TaD-CS ----CCTCCTTTTCAAAAAAAAATCAGAGGAATGATGATATGACTCAGTGAGACGCAAGT

* * *** * ** ************* ************** *****

TaB-CS CTTGCATACCTTGTACTGAT-AAATACCATGTGGCTGGCCCTCATGTGGTCTTCACAGAC

TaA-CS CTTGCATACCTTGTACAAAT-AGAAACCATGTGGCTGGCCCTCATGTGCTCTTCACAGAC

TaD-CS CTTGCATACCTTGTACTAATAAAATACCATGTGGCTGGCCCTCATGTGGCCTTCACAGAC

**************** ** * * *********************** **********

TaB-CS CTTGGCGAGCGCATTCAGCAGCCTGGAGGGAATGACCTGCAACAAAGTAGAAGGTGCAAT

TaA-CS CTTGTCGAGCGCATTCAGCAGCCTGGAGGGAATGACCTGCAACAAAGTAGAAGGTGCAAT

TaD-CS CTTGGCGGGCGCATTCAGCAGCCTGGAGGGAATGACCTGTAACAGAGTAGAAGGTGCGAT

**** ** ******************************* **** ************ **

TaB-CS CTACGCCTTCCCACGGATCCACCTCCCTGCAGCGGCGATCAAAGCCGCCAAGGCCGAGGG

TaA-CS CTACGCCTTCCCACGGATCCACCTCCCTGCAGCGGCGATCAAAGCCGCCAAGGCCGAGGG

TaD-CS CTACGCCTTCCCACGGATCCACCTCCCTGCAGCGGCGATCAAAGCCGCCAAGGCCGAGGG

************************************************************

TaB-CS CATGTCTCCAGACATGTTCTACGCGTGCCGCCTTCTCAACGCCACCGGGATCGCCGTTGT

TaA-CS CGTGTCCCCAGACATGTTCTACGCGTGCCGCCTTCTCGACGCCACCGGGATCGCCGTCGT

TaD-CS CATGTCTCCAGACTTGTTCTACGCGTGCCGCCTTCTCGACGCCACTGGGATTGCCGTCGT

* **** ****** *********************** ******* ***** ***** **

TaB-CS CCCTGGCTCTGGATTCCACCAGGTCAGTCCTTTTTTTGTTTTATTATTTATCAGGACTGC

TaA-CS CCCTGGCTCTGGATTCCACCAGGTCAGTCCTTTTTTGTTTTATCTATTTATCAGGACTGC

TaD-CS CCCTGGCTCTGGATTCCACCAGGTCAGTCCTTTTTTGTTTTATTTATTTATCAGGACTGC

************************************ *** ****************

TaB-CS ATCTGTAAGGGAAAAAAAATATAAGGAAGATGGTGTTGTCTAGTACTTGAAGTTGTTTCC

TaA-CS ATCTGGAAGGGGAAAAAAATAGAAGGAAGATGGTGTTGTCTAGTACTTGAAGTTGTTTCC

TaD-CS ATCTGGAAGGGAAAAA----------AAGAGGGTGTTGTCTAGTACTTGAAGTTGTTTCC

***** ***** **** **** *****************************

TaB-CS ATGCACAT----------------------------------------------------

TaA-CS ATGCACATGTACTCCTTCCGTTCCTAAATATAAGTCCTTTTAGAGATTCCACTATGGACT

TaD-CS ATGCACAT----------------------------------------------------

********

TaB-CS ------------------------------------------------------------

TaA-CS ACATACGGATGCATATAGACATATTTTAGAGTACGGATTCACTCATTTTGCTCCATATGT

TaD-CS ------------------------------------------------------------

TaB-CS -------------------------------------------------GCATATGTTAC

TaA-CS AGTTCCTTATAGGAATCTCTAAAAAGACTTATATTTAGAAATGGAGGACGTATATGTTAC

TaD-CS -------------------------------------------------GTATATGTTGC

* ******* *

TaB-CS AAGATATTATTAGTTGCCTGACTGTGGTGGCCTTTGATCTTGTTGCAAACGACTTCCATG

TaA-CS AAGATGTTGTTAGTTGCCTGACTCTGGTGGCCTTTGATCTTGTTGCAAACGACTTCCATG

TaD-CS AAGATGTTGTTAGTTGCCTGACTGTGGTGGCCTTTGATCTTGTTTCAAACGACTTCCATG

***** ** ************** ******************** ***************

TaB-CS GACGCATCAAAGGTGTCTGGGCGCAACAAGGCCACCGGGACATGGCATATCCGGTGCACG

TaA-CS GACGCAACAAAGGTGTCTGGGCGCAACAAGGCCACCGGGACATGTCATATCCGGTGCACG

TaD-CS GACGCAACAAAGGTGTCTGGGCGAAACAAGGCCACCGGGACATGGCATATCCGGTGCACG

****** **************** ******************** ***************

TaB-CS ATCCTCCCCGGCGAGGACAAGATCAAGGCGATGATCCCGCGCCTCAAGGAGTTCCACGAG

TaA-CS ATCCTCCCGGGCGAGGAGAAGATCAAGGAGATGATCCCGCGCCTCAAGGAGTTCCACGAG

TaD-CS ATCCTCCCCGGCGAGGACAAGATCAAGGTAATGATCCCGCGCCTCAAGGAGTTCCACGAG

******** ******** ********** ******************************

TaB-CS TCCTTCATGAACGAGTTCCGCAACCGAAGC**TGA**TGCGCACTTTCACCTACACAAGCCACA

TaA-CS TCCTTCATGAACGAGTTCCGCGACCGAAGC**TGA**TGTGCATCTTCAGCTACACAAGCCACA

TaD-CS TCCTTCATGAACGAGTTCCGTGACCGAAGC**TGA**TGCGCATCTTCAGCTACACAAGCCACA

******************** ************* *** **** **************

TaB-CS GGCCGGTCAGAGCTCCGAA---GGTGTGATCTTCA-----CTACCTTGTAAATTAAACCT

TaA-CS GGCCGGTCAGAGCCCTGAAGGCGGTGTGATCTACC-----CTACCCTGTAAATTAAGCCT

TaD-CS GGCCGGTCAGAGCCCCGAAGGCGGTGTGATCTTCACTACCCTACCCTGTAAATTAAGCCT

************* * *** ********** * ***** ********** ***

TaB-CS GTGTTGTACTGCCAATAAATAAACTTTCACTACCCTGTAAATTAAGCCTGTGTTGTACTA

TaA-CS GTGTTGTACTACCAATAAATAAACTTTCACTACCCTGTAAATTAAGCCTGTGTTGTACTG

TaD-CS GTGTTGTACTGCCAATAAATAAACTTTCACTACCCTGTAAATTAAGCCTGTGTTGTACTA

********** ************************************************

TaB-CS CCAATAAATAAACTTTGGCAGCTGTGTGTT

TaA-CS CAAATAAATAAACTTTGCCAGCA-------

TaD-CS CCAATAAATAAACTTTG-------------

* ***************
