# Supplementary material for: Sequence differences in the seed dormancy gene Qsd1 among various wheat genomes
Source: BMC Genomics. 2017 Jun 29;18:497. doi: 10.1186/s12864-017-3880-6 (PMC5492916; doi:10.1186/s12864-017-3880-6)
Supplement: Supplementary file 2 — Nucleotide sequences of Qsd1 orthologous loci in the sub-genomes of cv. Chinese Spring (TaA-CS, TaB-CS, and TaD-CS). The cDNA sequences are underlined. Start and stop codons are shown in bold. Introns are shown in standard font. b Nucleotide sequences of Qsd1 orthologs in the A sub-genomes of cv. Chinese Spring (TaA-CS), Triticum boeoticum (TbA), and T. monococcum (TmA). The cDNA sequences are underlined. Polymorphisms between the A sub-genome vs. diploid wheat and within diploid wheat accessions are highlighted in green and purple, respectively. Start and stop codons are shown in bold. The primer positions used for sequencing are highlighted in gray and marker names are shown in parentheses. (ZIP 43 kb) [file 12864_2017_3880_MOESM2_ESM.zip › Supplementary Material 3 (Figure S3b).docx]

**Figure S3b.** Nucleotide sequences of *Qsd1* orthologs in the A sub-genomes of cv. Chinese Spring (TaA-CS), *Triticum boeoticum* (TbA), and *T. monococcum* (TmA). The cDNA sequences are underlined. Polymorphisms between the A sub-genome vs. diploid wheat and within diploid wheat accessions are highlighted in green and purple, respectively. Start and stop codons are shown in bold. The primer positions used for sequencing are highlighted in gray and marker names are shown in parentheses.

TaA-CS -----------------------------------------------------------

TbA ACTGTCGCAATACTGCTTCGGAAAAGCATGACAGCCGAGCCCTAGGTCGGACGGACGCG

TmA ACTGTCGCAATACTGCTTCGGAAAAGCATGACAGCCGAGCGCTAGGTCGGACGGACGCG

(1)

TaA-CS ------------------------------------------------------------

TbA CGGCAGGGGACTATCTAGGAATATCATAAATATCTAGATAAAATAGATGATGTGGCAAGC

TmA CGGCAGGGGACTATCTAGGAATATCATAAATATCTAGATAAAATAGATGATATAGCAAGC

TaA-CS ------------------------------------------------------------

TbA AATAAATGAAAAAAAA------TGGTAACATAGCTAGCTACTAATAGTGATAACATCACA

TmA AATAAATGAAAAAAAAGAAAAATGGTAACATAGCTAGCTACTAATAGTGATAACATCACA

TaA-CS ------------------------------------------------------------

TbA CATATCAAAGCAAAATGTGGCTATAGCCTAATAAATGAAAGATTGCATGTTATCACACAT

TmA CATATCAAAGTAAAATGTGGCTATAGCCTAATAAATGAAAGATTGCATGTTACCACACAT

TaA-CS ------------------------------------------------------------

TbA ATATTACTCCCTACTATAAAAATAGTAACATAAACTAGTAATATGTCGATGTTACTACTC

TmA ATATTACTCCCTACTATAGAAATAGTAACATAAACTAGTAACATGTCGATGTTACTACTC

TaA-CS ------------------------------------------------------------

TbA TATGTTACTATCCATTGTGGCTAGTCTTACTGTTGCTTTACACGCCGCCGAAGGCCCAGA

TmA TATGTTACTATCCATTGTGGCTAGTCTTA----TGCTTTACACGCCGCCGAAGGCCCAGA

TaA-CS ------------------------------------------------------------

TbA CTCAGAGGCTCGTTCCCACGCTGGACGTGGCAGCCGCGGCCTGCCAAGCAGTGCCCACCG

TmA CTCAGAGGCTCGTTCCCACGCTGGACGTGGCAGCCGCGGCCTGCCAAGCAGTGCCCACCG

TaA-CS --------------------------------------------------CAATCACTCA

TbA CCACCGCCTAAGCTCCCCCCTTGACCCGGGCTCTGCCACCCAGCACGGCACAATCACTCA

TmA CCACCGCCTAAGCTCCCCCCTTGACCCGGGCTCTGCCACCCAGCACGGCACAATCACTCA

**********

TaA-CS CTCTCTGCCTCCTCCCATTTCCTCTGCGCCACCTTAGCCAAGCTCTCCTCCTTCCTTCCC

TbA CTCTCTGCCTCCTCCCATCTCCTCTGCGCCACCTTAGCCAAGCTCTCCTCCTTCCTTCCC

TmA CTCTCTGCCTCCTCCCATCTCCTCTGCGCCACCTTAGCCAAGCTCTCCTCCTTCCTTCCC

****************** *****************************************

TaA-CS CGCGCGCGCTCTCTCCTCGCCGGCGCCGCCCCGCGCC**ATG**TCGTACAACAAGACCGCCTC

TbA CGCGCGCGCTCTCTCCTCGCCGGCGCCGCCCCACGCC**ATG**TCGTACAACAAGACCGCCTC

TmA CGCGCGCGCTCTCTCCTCGCCGGCGCCGCCCCACGCC**ATG**TCGTACAACAAGACCGCCTC

******************************** ***************************

(2)

TaA-CS CATCACCGCCGAGACCATAAACCCCAAGGTACCTGACGGACGCCTCCTCATCCGGTGCTT

TbA CATCACCGCCGAGACCATAAACCCCAAGGTACCTGACGGACGCCTCCTCATCCGGTGCTT

TmA CATCACCGCCGAGACCATAAACCCCAAGGTACCTGACGGACGCCTCCTCATCCGGTGCTT

************************************************************

TaA-CS GCCCTGATTCACGTACGCCAAACCTGACTTGATTCTCCATGTTTTCCTTTCTTCCTCGCT

TbA GCCCTGATTCACGTACGCCAAACCTGACTTGATTCTCCATGTTTTCCTTTCTTCCTCGCT

TmA GCCCTGATTCACGTACGCCAAACCTGACTTGATTCTCCATGTTTTCCTTTCTTCCTCGCT

************************************************************

(3)

TaA-CS CACAGGTGAAGATCTTCGACTACGAGCCCTGCGGGGAGATCGCCAGGCACGCAGAGGTAA

TbA CACAGGTGAAGATCTTCGACTACGAGCCCTGCGGGGAGATCGCCAGGCACGCAGAGGTAA

TmA CACAGGTGAAGATCTTCGACTACGAGCCCTGCGGGGAGATCGCCAGGCACGCAGAGGTAA

************************************************************

TaA-CS CAGCAACAATTCTACCTGCACTCACCCTCGCCGTTCAACTGTCCATGTATGTATGTATGT

TbA CAGCAACAATTCTACCTGCACTCACCCTCGCCGTTCAACTGTCCATGTATGTATGTATGT

TmA CAGCAACAATTCTACCTGCACTCACCCTCGCCGTTCAACTGTCCATGTATGTATGTATGT

************************************************************

TaA-CS GTCGCAGTAGTCCAGTCAGACTGTCTGTCTATGGACACAGTCAGTGGCAGTGGGTTACCC

TbA GTCGCAGTAGTCCAGTCAGGCTGTCTGTCTATCGACACAGTCAGTGGCAGTGGGTTACCC

TmA GTCGCAGTAGTCCAGTCAGGCTGTCTGTCTATCGACACAGTCAGTGGCAGTGGGTTACCC

******************* ************ ***************************

TaA-CS CTGACCTAGTAGTACCCGGGTGCGCAAGCTAACTAAAGAAATGACTTCTGTAAACTGAAA

TbA CTGACCTAGTAGTACCCGGGTGCGCAAGCTAACTAAAGAAATGACTTCTGTAAACTGAAA

TmA CTGACCTAGTAGTACCCGGGTGCGCAAGCTAACTAAAGAAATGACTTCTGTAAACTGAAA

************************************************************

TaA-CS CCAAGTAGTGCACAAGCCAACTAAAGAAATGACTTCTGTAAACTGAAACTAAGAGCTAGA

TbA CCAAGTAGTGCACAAGCCAACTAAAGAAATGACTTCTGTAAACTGAAACTAAGAGCTAGA

TmA CCAAGTAGTGCACAAGCCAACTAAAGAAATGACTTCTGTAAACTGAAACTAAGAGCTAGA

************************************************************

TaA-CS CTGCATTTTGCACTACAGCCAGTTCAGATGATGCATCCAACTTTGATCTTTGGTATCATC

TbA CTGCATTTTGCACTACAGCCAGTTCAGATGATGCATCCAACTTTGATCTTTGGTATCATC

TmA CTGCATTTTGCACTACAGCCAGTTCAGATGATGCATCCAACTTTGATCTTTGGTATCATC

************************************************************

TaA-CS TGACATTCCTGGCCCATAAATCTGCAAAAGCACTGGTTTGACTGTACAAATTTTCTTTCG

TbA TGACATTCCTGGCCCATAAATCTGCAAAAGCACTGGTTTGACTGTACAAATTTTCTTTCG

TmA TGACATTCCTGGCCCATAAATCTGCAAAAGCACTGGTTTGACTGTACAAATTTTCTTTCG

************************************************************

TaA-CS GCATATGCCGGTTTTCGTGTTATCCAGCGGTTGGAGCAGGAGATGGAGAAGAGCCCCGGT

TbA GCATATGCCGGTTTTCGTGTTATCCAGCGGTTGGAGCAGGAGATGGAGAAGAGCCCCGGT

TmA GCATATGCCGGTTTTCGTGTTATCCAGCGGTTGGAGCAGGAGATGGAGAAGAGCCCCGGT

************************************************************

TaA-CS TCTCGCCCTTTCCCAGAGGTGCGTCTTTGTTTACATACAATGTATTCAATCCTGGCCAAA

TbA TCTCGCCCTTTCCCAGAGGTGCGTCTTTGTTTACATATAATGTATTCAATCCTGGCCAAA

TmA TCTCGCCCTTTCCCAGAGGTGCGTCTTTGTTTACATATAATGTATTCAATCCTGGCCAAA

************************************* **********************

(4)

TaA-CS ATCACTAGTCAATGATGATAAGTTGCTTCCTTGCAGATAACATACTGCAACCTTGGGAAC

TbA ATCACTAGTCAATGATGATAAGTTGCTTCCTTGCAGATAACATACTGCAACCTTGGGAAC

TmA ATCACTAGTCAATGATGATAAGTTGCTTCCTTGCAGATAACATACTGCAACCTTGGGAAC

************************************************************

TaA-CS CCCCAGGCTCTCGGCCAGCGACCCATAACCTTCTTCCGTGAGGTTACAACCTTTAATCAC

TbA CCCCAGGCTCTCGGCCAGCGACCCATAACCTTCTTCCGTGAGGTTACAACCCTTAATCAC

TmA CCCCAGGCTCTCGGCCAGCGACCCATAACCTTCTTCCGTGAGGTTACAACCCTTAATCAC

*************************************************** ********

TaA-CS ACTCTCCAAAAAATATTAATCCGGGAGGCATTAATTGCTGTCTTATGCCTCAAATATCAT

TbA ACTCTCCAAAAAATATTAATCCGGGAGCCATTAATTGCTGTCTTATGCCTCAAATATCAT

TmA ACTCTCCAAAAAATATTAATCCGGGAGCCATTAATTGCTGTCTTATGCCTCAAATATCAT

*************************** ********************************

TaA-CS CATCTGCCTTCAGGTTCTTTCCCTGTGCGACAATCCAGCTCTCCTGCGCAGGGATGAAAC

TbA CATCTGCCTTCAGGTTCTTTCCCTGTGCGACAATCCAGCTCTCCTGCGCAGGGATGAAAC

TmA CATCTGCCTTCAGGTTCTTTCCCTGTGCGACAATCCAGCTCTCCTGCGCAGGGATGAAAC

************************************************************

TaA-CS TCGTATGCTATTCAGGTTTGTTGGGCTTAAATAATACTGTACTGCTAGCTCAAAGTCTGT

TbA TCGTATGCTATTCAGGTTTGTTGG-----------------------GCTCAAAGTCTGT

TmA TCGTATGCTATTCAGGTTTGTTGG-----------------------GCTCAAAGTCTGT

************************ *************

TaA-CS TTTCCCATCTCTCACAATTTTCTCTTCAGAAGAAAAGCAAATTGCCATTGATGCATTTGC

TbA TTACCCATCTCTCACAATTTTCTCTTCAGAAGAAAAGCAAATTGCCATTGATGCATTTGC

TmA TTACCCATCTCTCACAATTTTCTCTTCAGAAGAAAAGCAAATTGCCATTGATGCATTTGC

** *********************************************************

(5)

TaA-CS TTTCAGTTCTAGCAGAAAAGTTGCTAGTAGTATCATGCACCATCTTGCTGTGACGATTTG

TbA TTTCAGTTCTAGCAGAAAAGTTGCTAGTAGTATCATGCACCATCTTGCTGTGACGATTTG

TmA TTTCAGTTCTAGCAGAAAAGTTGCTAGTAGTATCATGCACCATCTTGCTGTGACGATTTG

************************************************************

TaA-CS ATTCGTCGAAACCTGACCAGTATTGTTTCAACCTGCTATTACAGCCCATGTGCCATAAAT

TbA ATTCGTCGAAACCTGACCAGTATTGTTTCAACCTGCTATTACAGCCCATGTGCCATAAAT

TmA ATTCGTCGAAACCTGACCAGTATTGTTTCAACCTGCTATTACAGCCCATGTGCCATAAAT

************************************************************

(44160_L)

TaA-CS AGAGCGCGGAAGATTATTGAGTCCATGCCTGGCAGAAACTCCGGTGCATATACTAACAGT

TbA AGAGCGCGGAAGATTATTGAGTCCATGCCTGGCAGAAACTCCGGTGCATATACTAACAGT

TmA AGAGCGCGGAAGATTATTGAGTCCATGCCTGGCAGAAACTCCGGTGCATATACTAACAGT

************************************************************

TaA-CS CAGGTATTACATTGGAATACCAAGATAATCTTACAACCAGGTGCCTGATGTTTCGACTTT

TbA CAGGTATTACATTGGAATACCAAGATAATCTTACAACCAGGTGCCTGATGTTTCGACTTT

TmA CAGGTATTACATTGGAATACCAAGATAATCTTACAACCAGGTGCCTGATGTTTCGACTTT

************************************************************

TaA-CS TGGGGCGGTGCCGCATTAGTGCACCAACACCCTGGTCTTGGTGTTGTTGTCTTGTCCGGC

TbA TGGGGCGGTGCCGCATTAGTGCACCAACACCCTGGTCTTGGTGTTGTTGTCTTGTCCGGC

TmA TGGGGCGGTGCCGCATTAGTGCACCAACACCCTGGTCTTGGTGTTGTTGTCTTGTCCGGC

************************************************************

TaA-CS GATGATAATATTTTTCCCATACTATTCTGCACCACGTTGAGACACTTATTTTGGGACGGA

TbA GATGATAATATTTTTCCCATACTATTCTGCACCACGTTGAGACACTTATTTTGGGACGGA

TmA GATGATAATATTTTTCCCATACTATTCTGCACCACGTTGAGACACTTATTTTGGGACGGA

************************************************************

TaA-CS CGGAGTATTTAGGAACGGAGACAGTAATATTTTTTTCCATATTATTCTGCACCACGTTGT

TbA CGGAGTATTTAGGAACGGAGACAGTAATATTTTTTTCCATATTATTCTGCACCACGTTGT

TmA CGGAGTATTTAGGAACGGAGACAGTAATATTTTTTTCCATATTATTCTGCACCACGTTGT

************************************************************

TaA-CS TGTAAACTCCTCTAACATGATCAGGGAATCAGAAGTTTGCGCGAAGCAGTCGCAAGTGGA

TbA TGTAAACTCCTCTAACATGATCAGGGAATCAGAAGTTTGCGCGAAGCAGTCGCAAGTGGA

TmA TGTAAACTCCTCTAACATGATCAGGGAATCAGAAGTTTGCGCGAAGCAGTCGCAAGTGGA

************************************************************

(6)

TaA-CS ATCGCTGCAAGAGATGGTTTTCCATCAAGACCAGAAGACATCTTTCTGACAGATGGAGCG

TbA ATCGCTGCAAGAGATGGTTTTCCATCAAGACCAGAAGACATCTTTCTGACAGATGGAGCG

TmA ATCGCTGCAAGAGATGGTTTTCCATCAAGACCAGAAGACATCTTTCTGACAGATGGAGCG

************************************************************

TaA-CS AGTTCAGCCGTAATACTCTACATTCAACAGCTCCATCACATCAATGCACTTCGTCTATCT

TbA AGTTCAGCCGTAATACTCTACATTCAACAGCTCCATCACATCAATGCACTTCGTGTATCT

TmA AGTTCAGCCGTAATACTCTACATTCAACAGCTCCATCACATCAATGCACTTCGTGTATCT

****************************************************** *****

(7)

TaA-CS CGACGTGCTAAATTGGGCTGCATTTTTCATTTCTTCCTTCCAGATTAATTTGAGTATGCA

TbA CGACGTGCTAAATTGGGCTGCATTTTTCATTTCTTCCTTCCAGATTAATTTGAGTATGCA

TmA CGACGTGCTAAATTGGGCTGCATTTTTCATTTCTTCCTTCCAGATTAATTTGAGTATGCA

************************************************************

TaA-CS GATACTCATTAGGTCCCAAGAAGATGGCGTTTTATGCCCTTTACCTGAATATCCGTTATA

TbA GATACTCATTAGGTCCCAAGAAGATGGCGTTTTATGCCCTTTACCTGAATATCCGTTATA

TmA GATACTCATTAGGTCCCAAGAAGATGGCGTTTTATGCCCTTTACCTGAATATCCGTTATA

************************************************************

TaA-CS CTCGGCGTCCATTATACTTCATGGTGGGACTATGGTATGGTGCTAGCTTTATGAAATAAT

TbA CTCGGCGTCCATTATACTTCATGGTGGGACTATGGTATGGTGCTAGCTTTATGAAATAAT

TmA CTCGGCGTCCATTATACTTCATGGTGGGACTATGGTATGGTGCTAGCTTTATGAAATAAT

************************************************************

TaA-CS TGATCGGCGGTCGCAATTCTTCAGTACTTAATTTGTTCATTGCATCTCAAACCTATAGAT

TbA TGATCGGCGGTCGCAATTCTTCAGTACTTAATTTGTTCATTGCATCTCAAACCTATAGAT

TmA TGATCGGCGGTCGCAATTCTTCAGTACTTAATTTGTTCATTGCATCTCAAACCTATAGAT

************************************************************

TaA-CS CAGATTCCTGACATTTTTACATAAAAAGTGTAAAGGACTGCTCATTTTATTACATGCAAG

TbA CAGATTCCTGACATTTTTACATAAAAAGTGTAAAGGACTGCTCATTTTATTACATGCAAG

TmA CAGATTCCTGACATTTTTACATAAAAAGTGTAAAGGACTGCTCATTTTATTACATGCAAG

************************************************************

TaA-CS AAATGACCAAGTTACTGAATTTGGTTTGTAGGTGCCATACAATCTTAGTGAGGACGGTGA

TbA AAATGACCAAGTTACTGAATTTGGTTTGTAGGTGCCATACAATCTTAGTGAGGACGGTGA

TmA AAATGACCAAGTTACTGAATTTGGTTTGTAGGTGCCATACAATCTTAGTGAGGACGGTGA

************************************************************

TaA-CS TTGGGGGCTTGAGATCTTCGAAGTAAAGAGGTGCTTGGAGGAGGCACGCATCGCAGGTTT

TbA TTGGGGGCTTGAGATCTTCGAAGTAAAGAGGTGCTTGGAGGAGGCACGCATCGCAGGTTT

TmA TTGGGGGCTTGAGATCTTCGAAGTAAAGAGGTGCTTGGAGGAGGCACGCATCGCAGGTTT

************************************************************

(8)

TaA-CS GACTGTTCGGGCTATGGTGATCATAAACCCCGGAAATCCGACGGGACAGGTACGCAAACA

TbA GACTGTTCGGGCTATGGTGATCATAAACCCCGGAAATCCGACGGGACAGGTACGCAAACA

TmA GACTGTTCGGGCTATGGTGATCATAAACCCCGGAAATCCGACGGGACAGGTACGCAAACA

************************************************************

TaA-CS GATATTTATGTACCTTGCAATACCATAAATGTGAATGCATGGGATAAAACAAAAAAATAT

TbA GATATTTATGTACCTTGCAATACCATAAATGTGAATGCATGGGATAAAACAAAAAAATAT

TmA GATATTTATGTACCTTGCAATACCATAAATGTGAATGCATGGGATAAAACAAAAAAATAT

************************************************************

TaA-CS CTAGAAGGTTCCCTAAAATACTGCTGAAACTCAACATCTGCAAGCTCTGCTGTAGGTACT

TbA CTAGAAGGTTCCCTAAAATACTGCTGAAACTCAACATCTGCAAGCTCTGCTGTAGGTACT

TmA CTAGAAGGTTCCCTAAAATACTGCTGAAACTCAACATCTGCAAGCTCTGCTGTAGGTACT

************************************************************

TaA-CS GTCTATCACCAACCAGGAGGAGATAGTAGAATTTTGTCGGAAAGAAGGTTTGGTTATGCT

TbA GTCTATCACCAACCAGGAGGAGATAGTAGAATTTTGTCGGAAAGAAGGTTTGGTTATGCT

TmA GTCTATCACCAACCAGGAGGAGATAGTACAATTTTGTCGGAAAGAAGGTTTGGTTATGCT

**************************** *******************************

TaA-CS TGCCGATGAGGTTTGCACTTGGTGAGCTGCAGTAGTTTCCTGACGCGGACGCGTGTGTAG

TbA TGCCGATGAGGTTTGCACTTGGTGAGCTGCAGTAGTTTTCTGACGCGGACGCGTGTGTAG

TmA TGCCGATGAGGTTTGCACTTGGTGAGCTGCAGTAGTTTTCTGACGCGGACGCGTGTGTAG

************************************** *********************

TaA-CS ACATCCTACATTTGTGTAAATGTATTTACAGGTATACCAAGATAACGTCTATGTGGAGGA

TbA ACATCCTACATTTGTGTAAATGTATTTACAGGTATACCAAGATAACGTCTATGTGGAGGA

TmA ACATCCTACATTTGTGTAAATGTATTTACAGGTATACCAAGATAACGTCTATGTGGAGGA

************************************************************

TaA-CS TAGGAAATTTCATTCTTTCAAGAAAGTAGCCAGATCACTTGGGTATGACGAGAATGACAT

TbA TAGGAAATTTCATTCTTTCAAGAAAGTAGCCAGATCACTTGGGTATGACGAGAATGACAT

TmA TAGGAAATTTCATTCTTTCAAGAAAGTAGCCAGATCACTTGGGTATGACGAGAATGACAT

************************************************************

TaA-CS CTCCATAGTGTCATTTCACTCAGTCTCAATGGGTAAACCGTTGTTCATCAGAAACATAGT

TbA CTCCATAGTGTCATTTCACTCAGTCTCAATGGGTAAACCGTTGTTCATCAGAAACATAGT

TmA CTCCATAGTGTCATTTCACTCAGTCTCAATGGGTAAACCGTTGTTCATCAGAAACATAGT

************************************************************

(9)

TaA-CS TCGTAGCCTCTGGAAAATAAGCCTTAGAGCAGAGACTAATGGTTATTTTTTCATCTTCTG

TbA TCGTAGCCTCTGGAAAATAAGCCTTGGAGCAGAGACTAATGGTTATTTTTTCATCTTCTG

TmA TCGTAGCCTCTGGAAAATAAGCCTTAGAGCAGAGACTAATGGTTATTTTTTCATCTT-TG

************************* ******************************* **

TaA-CS AACTTGACAGGGTTCTCTGGAGAATGTGGCAGAAGGGGAGGCTACATGGAGATATGTGGT

TbA AACTTGACAGGGTTCTCTGGAGAATGTGGCAGAAGGGGAGGCTACATGGAGATATGTGGT

TmA AACTTGACAGGGTTCTCTGGAGAATGTGGCAGAAGGGGAGGCTACATGGAGATATGTGGT

************************************************************

TaA-CS TTTGGAGATGATGTGATGGGTGAGATTCGCAAAGTGGCTTCCGTGACTCTTTGCCCCAAC

TbA TTTGGAGATGATGTGATGGGTGAGATTCGCAAAGTGGCTTCCGTGACTCTTTGCCCCAAC

TmA TTTGGAGATGATGTGATGGGTGAGATTCGCAAAGTGGCTTCCGTGACTCTTTGCCCCAAC

************************************************************

TaA-CS ATAGGTGGTCAAATTCTTACTAGCCTTGCTATGGATCCACCGAAGGTTTGTTCGTCCTGC

TbA ATAGGTGGTCAAATTCTTACTAGCCTTGCTATGGATCCACCGAAGGTTTGTTCGTCCTGC

TmA ATAGGTGGTCAAATTCTTACTAGCCTTGCTATGGATCCACCGAAGGTTTGTTCGTCCTGC

************************************************************

TaA-CS CCTTCTTGTGTTTGTTATAATTTATTTTAATTACTACAACCTCCGTCCCAAATTACTCGT

TbA CCTTCTTGTGTTTGTTATAATTTATTTTAATTACTACAACCTCCGTCCCAAATTACTCGT

TmA CCTTCTTGTGTTTGTTATAATTTATTTTAATTACTACAACCTCCGTCCCAAATTACTCAT

********************************************************** *

TaA-CS CTTAGATTTGTCTAGATACTGATGTATCTAACAGTAAAACGTGTCTGTATCTTTATCTAG

TbA CTTAGATTTGTCTAGATACTGATGTATCTAACAGTAAAACGTGTCTGTATCTGTATCTAG

TmA CTTAGATTTGTCTAGATACTGATGTATCTAACAGTAAAACGTGTCTGTATCTGTATCTAG

**************************************************** *******

TaA-CS ACAAATCTAAGACAAGTAATTCAGGACGGAGGGAGTATATCTTGTCTTATCCTGAAAGCA

TbA ACAAATCTAAGACAAGTAATTCAGGACGGAGGGAGTATATCTTGTCTTATCCTGAAAGCA

TmA ACAAATCTAAGACAAGTAATTCAGGACAGAGGGAGTATATCTTGTCTTATCCTGAAAGCA

*************************** ********************************

TaA-CS TTGTAAAGCTGCCATCCTTCATCGGTAATGCTAGCTGGTGAACCTTCGCTGGGAAGGAAC

TbA TTGTAAAGCTGCCATCCTTCATCGGTAATGCTAGCTGGTGAACCTTCGCTGGGAAGGAAC

TmA TTGTAAAGCTGCCATCCTTCATCGGTAATGCTAGCTGGTGAACCTTCGCTGGGAAGGAAC

************************************************************

TaA-CS TCCCAGTGAACACCCTCGCAGTCGTTTGATCAAGATCAAACGTGACAG-TTTTTTTGAAA

TbA TCCCAGTGAACACCCTCGCAGTCGTTTGATCAAGATCAAACGTGACAGTTTTTTTTGAAA

TmA TCCCAGTGAACACCCTCGCAGTCGTTTGATCAAGATCAAACGTGACAGTTTTTTTTGAAA

************************************************ ***********

TaA-CS ATTTTGCCTTTTCTAGGAAGGAATTGTCATACTAGTCTGAAGAAATTGCTGCCTCCCTAT

TbA ATTTTGCCTTTTCTAGGAAGGAATTGTCATACTAGTCTGAAGAAATTGCTGCCTCCCTAT

TmA ATTTTGCCTTTTCTAGGAAGGAATTGTCATACTAGTCTGAAGAAATTGCTGCCTCCCTAT

************************************************************

TaA-CS AACTAAAATTGCATGGAAATCATACGCAAATGCCAT-CCCCCCCAACGAACCTTCGCTAG

TbA AACTAAAATTGCATGGATATCATACGCAAATGCCATCCCCCCCCAACGAACCTTAACTAG

TmA AACTAAAATTGCATGGATATCATACGCAAATGCCAT-CCCCCCCAACGAACCTTCGCTAG

***************** ****************** ***************** ****

TaA-CS CTATTGAAGTCCTTACATGACAGTACCCAAAAAATGTCAAAAAGATTATAATTATTTTCA

TbA CTATTGAAGTCCTTACATGACAGTACCCAAAAAATGTCAAAAAGATTATAATTATTTTCA

TmA CTATTGAAGTCCTTACATGACAGTACCCAAAAAATGTCAAAAAGATTATAATTATTTTCA

************************************************************

TaA-CS GCAAAAAAAATCCTTATTGAC-TTTTTTAAATAAGTCCTTATTGACTTTTGCTGCAAGTT

TbA GCAAAAAAAATCCTTATTGACTTTTTTTAAATAAGTCCTTATTGACTTTTGCTGCAAGTT

TmA GCAAAAAAAATCCTTATTGACTTTTTTTAAATAAGTCCTTATTGACTTTTGCTGCAAGTT

********************* **************************************

(42384_L)

TaA-CS TCTTGGGCTGTTTTTATTGGGTTATTATTAATGTAATTATTCCTACCATTCGCATACTTG

TbA TCTTGGGCTGTTTTTATTGGGTTATTATTAATGTAATTATTCCTACCATTCGCATACTTG

TmA TCTTGGGCTGTTTTTATTGGGTTATTATTAATGTAATTATTCCTACCATTCGCATACTTG

************************************************************

TaA-CS TAATCAAAATTGATTTGATGCAGCTTTGATGAGATTTTCGTTCTGGTTTTTAGAC-AAGA

TbA TAATCAAAATTGATTTGATGCAGCTTTGATGAGATTTTCGTTCTGGTTTTTAGACAAAGA

TmA TAATCAAAATTGATTTGATGCAGCTTTGATGAGATTTTCGTTCTGGTTTTTAGACAAAGA

******************************************************* ****

TaA-CS GGTTTTTGTGTCCTTTGCAATATTAGTTGCCCGTTGACCCTTGCTGCCTCAATTTTGTCA

TbA GGTTTTTGTGTCCTTTGCAATATTAGTTGCCCGTTGACCCTTGCTGCCTCAATTTTGTCA

TmA GGTTTTTGTGTCCTTTGCAATATTAGTTGCCCGTTGACCCTTGCTGCCTCAATTTTGTCA

************************************************************

TaA-CS CAAAATAGACAACCCCCTACCTAGAAAAGAATAGATATGTATGTGTTTGTTTCTACCAAA

TbA CAAAATAGACAACCCCCTACCTAGAAAAGAATAGATATGTATGTGTTTGTTTCTACCAAA

TmA CAAAATAGACAACCCCCTACCTAGAAAAGAATAGATATGTATGTGTTTGTTTCTACCAAA

************************************************************

TaA-CS CTCATCTTGATATTTGCAGCTGGGAGATGGTTGTTTTGAGAATTTTATGGCTGAAAAGGA

TbA CTCATCTTGATATTTGCAGCTGGGAGATGGTTGTTTTGAGAATTTTATGGCTGAAAAGGA

TmA CTCATCTTGATATTTGCAGCTGGGAGATGGTTGTTTTGAGAATTTTATGGCTGAAAAGGA

************************************************************

TaA-CS AGACATCCGTTTATCTCTCGCCAAGCGCGCCAAGGTATCCTCCTCCTACATACACATTGT

TbA AGACATCCGTTTATCTCTCGCCAAGCGCGCCAAGGTATCCTCCTCCTACATACACATTGT

TmA AGACATCCGTTTATCTCTCGCCAAGCGCGCCAAGGTATCCTCCTCCTACATACACATTGT

************************************************************

TaA-CS CAACAAGCACACCATCTTTCTTCTTCCTCTATCAGAGGAATGATGAAATGACTCAGTGAG

TbA CAACAAGCACACCATCTTTCTTCTTCCTCTATCAGAGGAATGATGAAATGACTCAGTGAG

TmA CAACAAGCACACCATCTTTCTTCTTCCTCTATCAGAGGAATGATGAAATGACTCAGTGAG

************************************************************

TaA-CS ACACAAGTCTTGCATACCTTGTACAAATAGAAACCATGTGGCTGGCCCTCATGTGCTCTT

TbA ACACAAGTCTTGCATACCTTGTACAAATAGAAACCATGTGGCTGGCCCTCATGTGCTCTT

TmA ACACAAGTCTTGCATACCTTGTACAAATAGAAACCATGTGGCTGGCCCTCATGTGCTCTT

************************************************************

TaA-CS CACAGACCTTGTCGAGCGCATTCAGCAGCCTGGAGGGAATGACCTGCAACAAAGTAGAAG

TbA CACAGACCTTGTCGAGCGCATTCAGCAGCCTGGAGGGAATGACCTGCAACAAAGTAGAAG

TmA CACAGACCTTGTCGAGCGCATTCAGCAGCCTGGAGGGAATGACCTGCAACAAAGTAGAAG

************************************************************

(41242_R)

TaA-CS GTGCAATCTACGCCTTCCCACGGATCCACCTCCCTGCAGCGGCGATCAAAGCCGCCAAGG

TbA GTGCAATCTACGCCTTCCCACGGATCCACCTCCCTGCAGCGGCGATCAAAGCCGCCAAGG

TmA GTGCAATCTACGCCTTCCCACGGATCCACCTCCCTGCAGCGGCGATCAAAGCCGCCAAGG

************************************************************

TaA-CS CCGAGGGCGTGTCCCCAGACATGTTCTACGCGTGCCGCCTTCTCGACGCCACCGGGATCG

TbA CCGAGGGCGTGTCCCCAGACATGTTCTACGCGTGCCGCCTTCTCGACGCCACCGGGATCG

TmA CCGAGGGCGTGTCCCCAGACATGTTCTACGCGTGCCGCCTTCTCGACGCCACCGGGATCG

************************************************************

(10)

TaA-CS CCGTCGTCCCTGGCTCTGGATTCCACCAGGTCAGTCCTTTTTTGTTTTATCTATTTATCA

TbA CCGTCGTCCCTGGCTCTGGATTCCACCAGGTCAGTCCTTTTTTGTTTTATCTATTTATCA

TmA CCGTCGTCCCTGGCTCTGGATTCCACCAGGTCAGTCCTTTTTTGTTTTATCTATTTATCA

************************************************************

TaA-CS GGACTGCATCTGGAAGGGGAAAAAAATAGAAGGAAGATGGTGTTGTCTAGTACTTGAAGT

TbA GGACTGCATCTGGAAGGGGAAAAAAATAGAAGGAAGATGGTGTTGTCT-----------T

TmA GGACTGCATCTGGAAGGGGAAAAAAATAGAAGGAAGATGGTGTTGTCT-----------T

************************************************ *

TaA-CS TGTTTCCATGCACATGTACTCCTTCCGTTCCTAAATATAAGTCCTTTTAGAGATTCCACT

TbA TGTTTCCATGCACATGTACTTCCTCCGTTCCTAAATATAAGTCCTTTTAGAGATTCCACT

TmA TGTTTCCATGCACATGTACTTCCTCCGTTCCTAAATATAAGTCCTTTTAGAGATTCCACT

******************** * *************************************

TaA-CS ATGGACTACATACGGATGCATATAGACATATTTTAGAGTACGGATTCACTCATTTTGCTC

TbA ATGGACTACATACGGATGTATATAGACATATTTTAAAGTACGGATTCACTCATTTTGCTC

TmA ATGGACTACATACGGATGTATATATACATATTTTAGAGTACGGATTCACTCATTTTGCTC

****************** ***** ********** ************************

TaA-CS CATATGTAGTTCCTTATAGGAATCTCTAAAAAGACTTATATTTAGAAATGGAGGACGTAT

TbA CGTATGTAGTTCCTTATAGGAATCTCTAAAAAGACTTATATTTAGAAACGGAGGACGTAT

TmA CGTATGTAGTTCCTTATAGGAATCTCTAAAAAGACTTATATTTAGAAACGGAGGACGTAT

* ********************************************** ***********

TaA-CS ATGTTACAAGATGTTGTTAGTTGCCTGACTCTGGTGGCCTTTGATCTTGTTGCAAACGAC

TbA ATGTTACAAGATGTTGTTAGTTGCCTGACTCTGGTGGCCTTTGATCTTGTTGCAAACGAC

TmA ATGTTACAAGATGTTGTTAGTTGCCTGACTCTGGTGGCCTTTGATCTTGTTGCAAACGAC

************************************************************

(11)

TaA-CS TTCCATGGACGCAACAAAGGTGTCTGGGCGCAACAAGGCCACCGGGACATGTCATATCCG

TbA TTCCATGGACGCAACAAAGGTGTCTGGGCGCAACAAGGCCACCGGGACATGTCATATCCG

TmA TTCCATGGACGCAACAAAGGTGTCTGGGCGCAACAAGGCCACCGGGACATGTCATATCCG

************************************************************

TaA-CS GTGCACGATCCTCCCGGGCGAGGAGAAGATCAAGGAGATGATCCCGCGCCTCAAGGAGTT

TbA GTGCACGATCCTCCCGGGCGAGGAGAAGATCAAGGAGATGATCCCGCGCCTCAAGGAGTT

TmA GTGCACGATCCTCCCGGGCGAGGAGAAGATCAAGGAGATGATCCCGCGCCTCAAGGAGTT

************************************************************

TaA-CS CCACGAGTCCTTCATGAACGAGTTCCGCGACCGAAGC**TGA**TGTGCATCTTCAGCTACACA

TbA CCACGAGTCCTTCATGAACGAGTTCCGCGATCGAAGC**TGA**TGTGCATCTTCAGCTACACA

TmA CCACGAGTCCTTCATGAACGAGTTCCGCGACCGAAGC**TGA**TGTGCATCTTCAGCTACACA

****************************** *****************************

(12)

TaA-CS AGCCACAGGCCGGTCAGAGCCCTGAAGGCGGTGTGATCTACCCTACCCTGTAAATTAAGC

TbA AGCCACAGGCCGGTCAGAGCCCTGAAGGCGGTGTGATCTACCCTACCCTGTAAATTAAGC

TmA AGCCACAGGCCGGTCAGAGCCCTGAAGGCGGTGTGATCTACCCTACCCTGTAAATTAAGC

************************************************************

TaA-CS CTGTGTTGTACTACCAATAAATAAACTTTCACTACCCTGTAAATTAAGCCTGTGTTGTAC

TbA CTGTGTTGTACTACCAATAAATAAACTTTCACTACCCTGTAAATTAAGCCTGTGTTGTAC

TmA CTGTGTTGTACTACCAATAAATAAACTTTCACTACCCTGTAAATTAAGCCTGTGTTGTAC

************************************************************

TaA-CS TGCAAATAAATAAACTTTGCCAGCA-----------------------------------

TbA TGCAAATAAATAAACTTAGCCAGCAGTGTGTTGGTCCTTCCCTGTAAATTAAGCCTGTGT

TmA TGCAAATAAATAAACTTAGCCAGCAGTGTGTTGGTCCTTCCCTGTAAATTAAGCCTGTGT

***************** *******

TaA-CS ------------------------------------------------------------

TbA TGCTTTAATACACTTTGGCTCTGCGAAGTGCAAACCTGGTATATCCACCTGCCTGTCTCA

TmA TGCTTTAATACACTTTGGCTCTGCGAAGTGCAAACCTGGTATATCCACCTGCCTGTCTCA

TaA-CS ------------------------------------------------------------

TbA TTGCCGCGATGATCATAGTTATATCGCGACGAAACTGGCAGTTCAATGTACACTGATTCT

TmA TTGCCGCGATGATCATAGTTATATCGCGACGAAACTGGCAGTTCAATGTACACTGATTCT

TaA-CS ------------------------------------------------------------

TbA GCACCTCAATTCACAACATCCAACATTCAGAAGATCTGAATTCCGAGACGACTAGAGCGA

TmA GCACCTCAATTCACAACATCCAACATTCAGAAGATCTGAATTCCGAGACGACTAGAGCGA

　 (13)

TaA-CS ----------

TbA GCGAAGGACA

TmA GCGAAGGACA
